# Four New Citrinin Derivatives from a Marine-Derived *Penicillium* sp. Fungal Strain

**DOI:** 10.3390/molecules18055723

**Published:** 2013-05-16

**Authors:** Mei Ling Wang, Chun Hua Lu, Qing Yan Xu, Si Yang Song, Zhi Yu Hu, Zhong Hui Zheng

**Affiliations:** 1 School of Life Sciences, Xiamen University, 422 South Siming Road, Xiamen, Fujian 361005, China; E-Mails: wml1211@126.com (M.L.W); xuqingyan@xmu.edu.cn (Q.Y.X); sysong@xmu.edu.cn (S.Y.S); huzhiyu@xmu.edu.cn (Z.Y.H); 2 Key Laboratory of Chemical Biology (Ministry of Education), School of Pharmaceutical Sciences, Shandong University, Jinan, Shandong 250012, China; E-Mail: ahua0966@sdu.edu.cn

**Keywords:** citrinin, *Penicillium* sp. ML226, new metabolites, marine fungus

## Abstract

Four new citrinin derivatives, including two citrinin dimers and two citrinin monomer derivatives, were isolated and identified from a marine-derived fungal strain *Penicillium* sp. ML226 along with six known related compounds. Their structures were elucidated by spectroscopic and chemical methods. The new compounds showed modest cytotoxic activity against HepG-2 cell line and weak antimicrobial activity against *Staphylococcus aureus*.

## 1. Introduction

**Figure 1:**
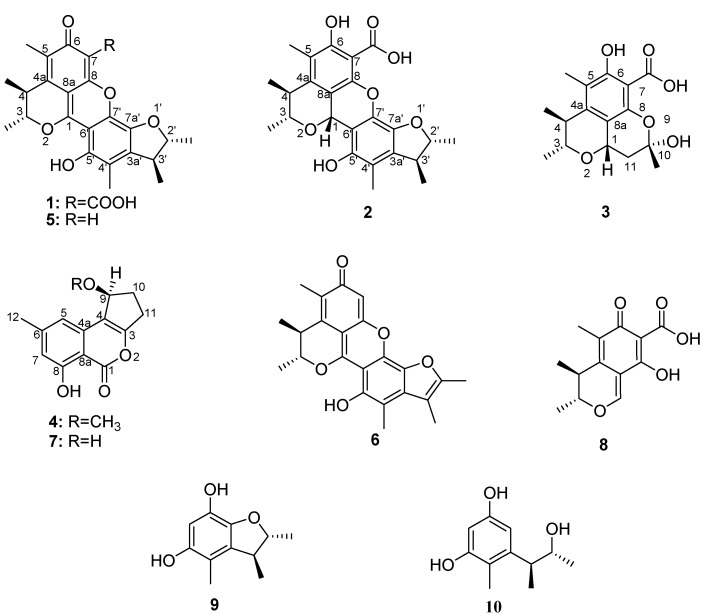
Structures of the isolated compounds **1**–**10**.

The search for new bioactive natural products is still the main way of discovering new drugs. Investigating the secondary metabolites of microorganisms isolated from specific ecological environments may increase the chance of finding compounds with novel skeletons and varied and unique bioactivities. It was reported that the specific situations that microorganisms live in might activate some silent genes and induce some unique biosynthetic pathways [1]. Marine microorganisms have attracted extensive attention in this context. Marine fungi are an important resource to find chemically and biologically diverse compounds due to their special living environment [2,3]. In order to search for new bioactive natural products, a marine-derived fungal strain, ML226, authenticated as *Penicillium* sp., was isolated from the Taiwan Strait, China. The EtOAc extract of *Penicillium* sp. ML226 exhibited cytotoxic and antimicrobial activity. Chemical investigation of the EtOAc extract of *Penicillium* sp. ML226 led to the isolation of two new citrinin dimers—penicitrinone E (**1**) and penicitrinol J (**2**)—two new citrinin monomer derivatives—penicitrinol K (**3**) and citrinolactone D (**4**)—together with six known compounds-penicitrinone A [4] (**5**), penicitrinone B [4] (**6**), citrinolactone B [5] (**7**), citrinin [6] (**8**), 2,3,4-trimethyl-5,7-dihydroxy-2,3-dihydrobenzofuran [7] (**9**) and phenol A [8] (**10**) (Figure 1). In this paper, we report the isolation and structural elucidation of compounds **1**–**10** and the cytotoxic and antimicrobial activity of **1**–**4**. They all showed weak cytotoxicity against HepG-2 cell line in the concentration of 10 μg/mL with inhibition rate from 6% to 30%. Compounds **2** and **3** showed weak antimicrobial activity against *Staphylococcus aureus*.

## 2. Results and Discussion

### 2.1. Structural Elucidation of Compounds

Penicitrinone E (**1**) was obtained as a red, amorphous powder. ${\left[\alpha \right]}_{D}^{25}$ 0 (0.002, MeOH). A molecular formula of C_24_H_24_O_7_ was assigned based on the interpretation of HRESIMS data at *m/z* 425.15965 [M + H]^+^ (calcd. 425.1600). The ^1^H-NMR data of **1** showed four tertiary methyl signals, two aromatic methyl signals, four sp^3^ methine protons (two oxygenated), and one hydroxyl proton (Table 1). The ^13^C-NMR and DEPT spectra for **1** displayed 24 carbon signals comprising four tertiary methyls, two aromatic methyls, four sp^3^ methines (two oxygenated), two carbonyl carbons, and 12 sp^2^ quaternary carbons (Table 1). Except for those of the benzopyran moiety, the NMR data were quite similar to those of **5** [4], indicating that they shared the same molecular skeleton. Compared with those of **5**, the NMR spectra of **1** exhibited an additional carboxyl group (*δ_c_* 165.4), two downfield shifts effect of C-1 (+4.6 ppm) and C-8 (+2.7 ppm) because of the inductive effect of the additional carboxyl group. The C-7 of **5** is a sp^2^ methine carbon but the C-7 of **1** is a sp^2^ quaternary carbon, indicating that the carboxyl group was linked to C-7.

**Table 1:** ^1^H- and ^13^C-NMR (600 and 150 MHz) data for compounds **1** and **2** (CDCl_3_, *δ* in ppm).

*No.*	1		2	
	*δ_H_ (mult., J [Hz], int.)*	*δ_c_*	*δ_H_ (mult., J [Hz], int.)*	*δ_c_*
1	-	161.1s	5.71 (s)	66.3d
3	5.17 (q, 6.7)	83.2d	4.10 (dq, 6.1, 6.8)	79.0d
3-CH_3_	1.49 (d, 6.7, 3H)	19.04q	1.47 (d, 6.8, 3H)	21.9q
4	3.29 (q, 7.2)	34.8d	3.03 (dq, 6.1, 7.0)	37.7d
4-CH_3_	1.38 (d, 7.2, 3H)	18.97q	1.34 (d, 7.0, 3H)	19.6q
4a	-	132.7s	-	144.7s
5	-	130.8s	-	120.6s
5-CH_3_	2.22 (s, 3H)	10.9q	2.21 (s, 3H)	11.2q
6	-	183.8s	-	161.9s
6-OH	-	-	12.52 (s)	-
7	-	103.1s	-	98.0s
7-COOH	-	165.4s	-	170.6s
8	-	160.9s	-	145.3s
8a	-	99.5s	-	108.9s
2′	4.77 (dq, 4.3, 6.4)	88.4d	4.56 (m)	88.3d
2′-CH3	1.47 (d, 6.4, 3H)	21.0q	1.36 (d, 6.5, 3H)	20.9q
3′	3.25 (dq, 4.3, 7.1)	45.0d	3.09 (m)	44.3d
3′-CH3	1.36 (d, 7.1, 3H)	18.82q	1.35 (d, 7.2, 3H)	19.3q
3a′	-	142.4s	-	133.0s
4′	-	118.2s	-	117.8s
4′-CH3	2.28 (s, 3H)	11.6q	2.18 (s, 3H)	11.6q
5′	-	147.2s	-	147.5s
5′-OH	8.25 (s)	-	7.92 (s)	-
6′	-	102.2s	-	105.3s
7′	-	136.2s	-	130.7s
7a′	-	139.2s	-	138.1s

^a^ The assignments were based on DEPT, ^1^H-^1^H COSY, HMQC, and HMBC experiments, and chemical shift values are in ppm relative to TMS. “-”: no signal.

**Figure 2:**
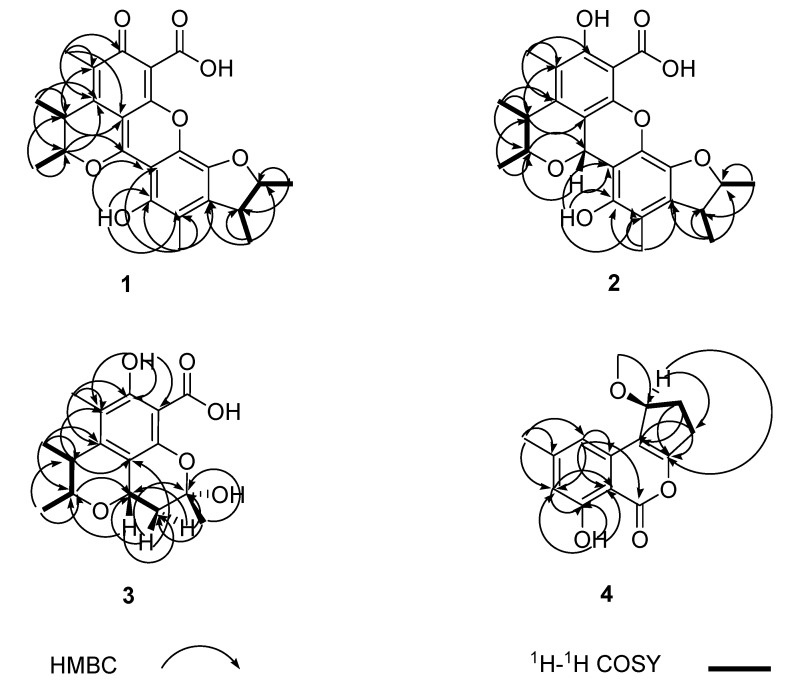
^1^H-^1^H COSY and key HMBC correlations of compounds **1**–**4**.

**Figure 3:**
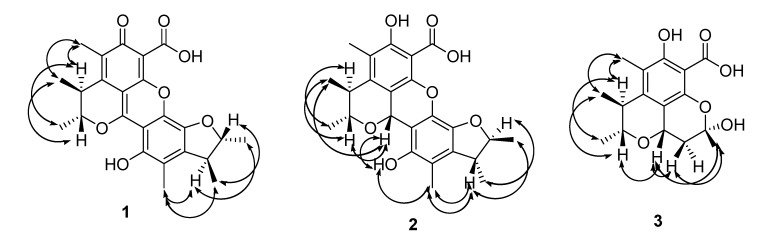
Key NOESY effects of compounds **1**–**3**.

This deduction was further supported by analyses of the 2D (HMQC, ^1^H-^1^H COSY and HMBC) NMR spectra (Figure 2). The relative configuration of the two methyl residues in the benzopyran moiety was determined as *trans* based on the NOESY correlation of 4-CH_3_ with 3-H and *J*^3,4^ (<0.5 Hz); and the relative configuration of the two methyl residues in the benzofuran moiety was determined as *trans* based on the NOESY correlation of 3′-CH_3_ with 2′-H and *J*^2′,3′^ (=4.3 Hz) (Figure 3).

Penicitrinol J (**2**) was isolated as a pale yellow, amorphous powder. ${\left[\alpha \right]}_{D}^{25}$ −30.0 (0.001, MeOH). The molecular formula of **2** was determined as C_24_H_26_O_7_ by HRESIMS at *m/z* 427.17601 [M + H]^+^ (calcd. 427.1757). The ^1^H-NMR data of **2** showed four tertiary methyl signals, two aromatic methyl signals, five sp^3^ methine protons (three oxygenated), and two hydroxyl protons (Table 1). The ^13^C-NMR and DEPT spectra for **2** displayed 24 carbon signals including four tertiary methyls, two aromatic methyls, five sp^3^ methines (three oxygenated), one carbonyl carbon, and 12 sp^2^ quaternary carbons (Table 1). The NMR data were quite similar to those of **1** except for those of the benzopyran moiety. Compared with those of **1**, the NMR spectra of **2** exhibited an additional oxygenated sp^3^ methine proton (*δ_H_* 5.71) and an additional oxygenated sp^3^ methine carbon (*δ_c_* 66.3), but missed one carbonyl carbon (*δ_c_* 183.8 in **1**). These indicated one of the two additional protons was linked to C-1, the other was the hydroxyl proton of 6-OH, which was further supported by the downfield shift effect of C-4a (+12 ppm) and the high-field shifts effect of H-3 (−1.07 ppm) and H-4 (−0.26 ppm) as a result of the missing of the double bond between C-1 and C-8a, and the 2D (HMQC, ^1^H-^1^H COSY and HMBC) NMR spectra (Figure 2). The NOESY correlation of 3-H with 4-CH_3_ and *J*^3,4^ (=6.1 Hz) established the *trans* of the two methyl residues in the benzopyran moiety; The NOESY correlation of 2′-H with 3′-CH_3_ and *J*^2,3^ (<0.5 Hz) demonstrated the *trans* of the two methyl residues in the benzofuran moiety; and the relative configuration of the 1-H and 3-H was determined as *cis* based on the NOESY correlations of 1-H with 3-H, 5′-OH and 4 -CH_3_ (Figure 3). 

**Figure 4:**
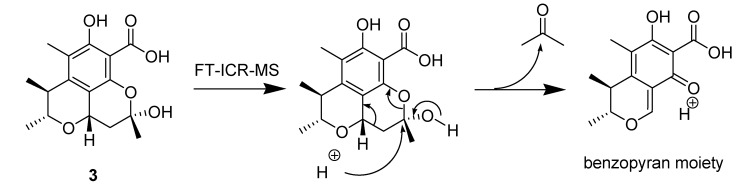
The benzopyran moiety of compound **3**.

Penicitrinol K (**3**) was isolated as a white, amorphous powder. ${\left[\alpha \right]}_{D}^{25}$ −125.2 (0.002, MeOH). The benzopyran moiety’s molecular formula of **3** was determined as C_13_H_14_O_5_ by HRESIMS at *m/z* 273.07299 [M + Na]^+^ (calcd. 273.0739) (Figure 4). 

The molecular formula of **3** was determined to be C_16_H_20_O_6_ by ESI-MS at *m/z* 331.2 [M + Na]^+^ and 291.4 [M − H_2_O + H]^+^. The ^1^H-NMR data of **3** showed three tertiary methyl signals, one aromatic methyl signal, one sp^3^ methylene signal, three sp^3^ methine protons (two being oxygenated), and one hydroxyl proton (Table 2). The ^13^C-NMR and DEPT spectra of **3** displayed signals for three tertiary methyls, one aromatic methyl, one sp^3^ methylene, three sp^3^ methines (two being oxygenated) and eight quaternary carbons (Table 2). Compared to those of **2**, compound **3** shared the same benzopyran moiety with **2** which was further supported by the 2D (HMQC, ^1^H-^1^H COSY and HMBC) spectra (Figure 2), and the NMR spectra of **3** exhibited a high-field shift effect of C-1 (−1.01 ppm). The ^1^H-^1^H COSY correlations between 1-H and 11-Ha, 1-H and 11-Hb, and key HMBC correlations from 1-H, 11-H and 10-CH_3_ to corresponding carbons indicated that C-11 was linked to C-1, C-10 was linked to C-11, and 10-CH_3_ was linked to C-10 (Figure 2). Finally C-10 was linked to C-8 via O and 10-OH was linked to C-10, which established by the molecular formula of **3**. The NOESY correlation of 3-H with 4-CH_3_ and *J*^3,4^ (=6.3 Hz) determined the *trans* of 3-CH_3_ and 4-CH_3_. The NOESY correlations between 1-H and 3-H and 1-H and 10-CH_3_ established the *cis* configurations of 1-H and 3-H and 1-H and 10-CH_3_ (Figure 3).

**Table 2:** The NMR data for compound **3** (CDCl_3_, *δ* in ppm).

*No.*	*δ_H_ (mult., J [Hz], int.)*	*δ_c_*
1	4.70 (dd, 6.1, 11.5)	66.2d
3	3.74 (dq, 6.3, 6.2)	78.8d
3-CH_3_	1.39 (d, 6.2, 3H)	21.6q
4	2.88 (dq, 6.3, 6.9)	38.1d
4-CH_3_	1.23 (d, 6.9, 3H)	19.3q
4a	-	145.8s
5	-	118.2s
5-CH_3_	2.14 (s, 3H)	11.1q
6	-	161.3s
6OH	12.16 (s)	-
7	-	97.6s
7-COOH	-	171.6s
8	-	146.9s
8a	-	111.7s
10	-	101.4s
10-CH_3_	1.87 (s, 3H)	29.3q
11a	2.53 (dd, 6.1, 12.8)	37.3t
11b	1.84 (dd, 11.5, 12.8)	

Citrinolactone D (**4**) was obtained as a white, amorphous powder. ${\left[\alpha \right]}_{D}^{25}$ +6.7 (0.004, MeOH). The molecular formula of **4** was determined as C_14_H_14_O_4_ by HRESIMS at *m/z* 269.07804 [M + Na]^+^ (calcd. 269.0790). The ^1^H-NMR data of **4** showed one aromatic methyl signal, one methoxyl group, two sp^3^ methylene signals, two aromatic protons, one oxygenated sp^3^ methine proton, and one hydroxyl proton (Table 3). The ^13^C-NMR spectrum of **4** displayed signals for two methyls (one of them oxygenated), two sp^3^ methylenes, one oxygenated sp^3^ methine, two sp^2^ methines, one carbonyl carbon, and 6 sp^2^ quaternary carbons (Table 3). The NMR data were quite similar to those of **7** [5]. By comparison with those of **7**, the NMR spectra of **4** exhibited an additional methoxyl group (*δ_H_* 3.50, *δ_c_* 57.3) attached C-9, and which was further supported by HMBC correlations from 9-OCH_3_ to C-9 (Figure 2). 

**Table 3:** The NMR data of compound **4** (CDCl_3_, *δ* in ppm).

*No.*	*δ_H_ (mult., J [Hz], int.)*	*δ_c_*
1	-	181.1s
3	-	173.9s
4	-	119.9s
4a	-	157.3s
5	6.71 (s)	107.6d
6	-	146.7s
7	6.63 (s)	112.6d
8	-	161.0s
8-OH	12.56 (s)	-
8a	-	109.0s
9	4.95 (d, 6.8)	79.4d
10a	2.35 (m)	27.6t
10b	2.17 (m)	
11a	3.22 (m)	30.2t
11b	2.81 (m)	
12	2.40 (s, 3H)	22.3q
9-OCH_3_	3.50 (s, 3H)	57.3q

### 2.2. Biosynthesis

**Scheme 1:**
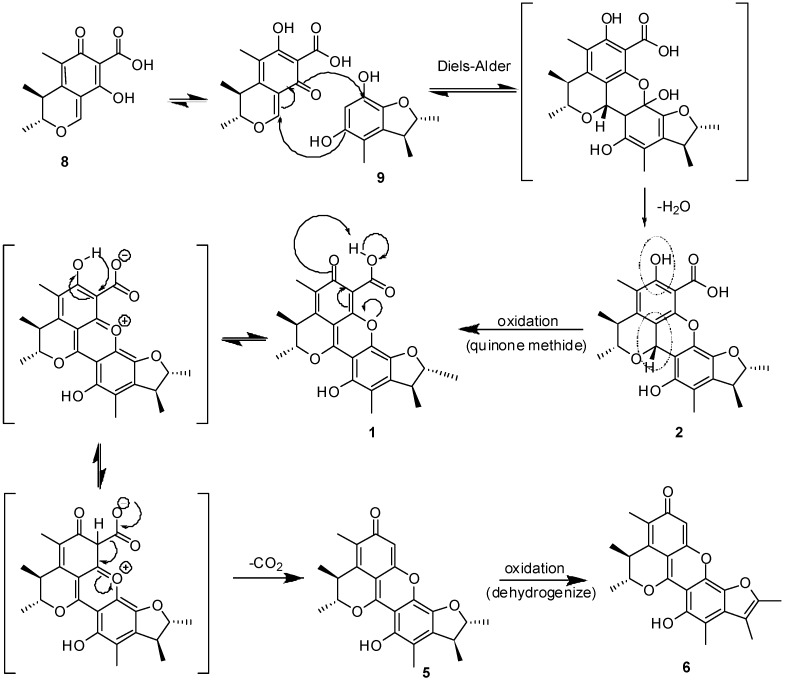
Postulated Biosynthesis of **1**, **2**, **5** and **6** resulting from **8** and **9**.

**Scheme 2:**
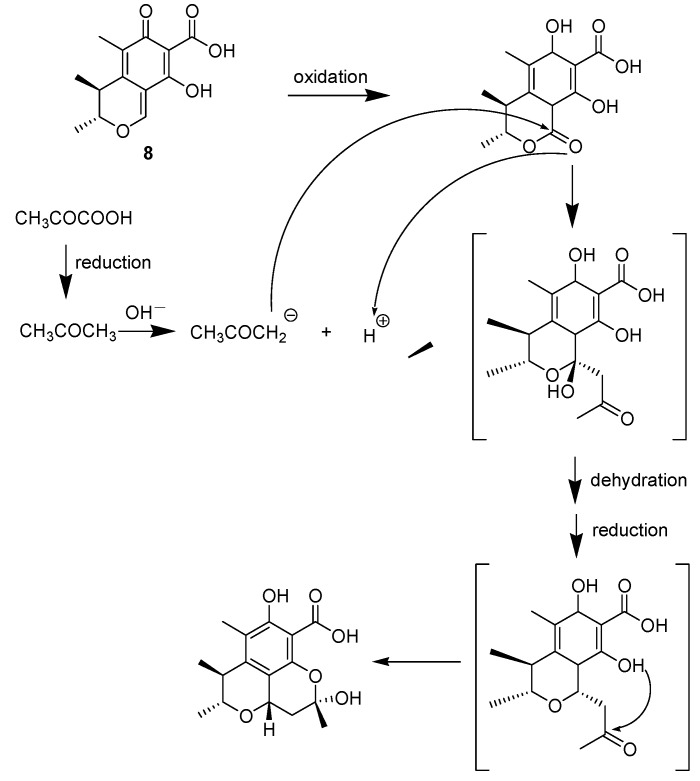
Postulated Biosynthetic Pathway of **3**.

These compounds likely have the same biogenetic origin via the polyketide pathway [9]. citrinolactone B (**7**), citrinin (**8**), 2,3,4-trimethyl-5,7-dihydroxy-2,3-dihydrobenzofuran (**9**), phenol A (**10**) are biosynthesized from acetyl coenzyme A. Citrinolactone D (**4**) is the result of the methylation of citrinolactone B at 9-OH (**7**). To explain the biogenetic origin of penicitrinone E (**1**), penicitrinol J (**2**) and penicitrinol K (**3**), a postulated biosynthetic pathway is proposed in Scheme 1 and Scheme 2. Penicitrinone E (**1**) is postulated to derive from the oxidation of penicitrinol J (**2**), which results from the Diels-Alder reaction of **8** with **9** and then undergoes dehydration. Subsequent decarboxylation and the following dehydrogenation of penicitrinol J (**2**), leads successively to penicitrinone A (**5**) and penicitrinone B (**6**) [10] (Scheme 1). Oxidation of citrinin (**8**) forms dihydrocitrinone [11]. After undergoing aldol condensation of the carbonyl group at C-1 of dihydrocitrinone with an acetone anion, which is biosynthesized from pyruvic acid via enzymatic reduction [12], dihydrocitrinone changes to an intermediate structure. Further dehydration, reduction and the following aldol condensation of the intermediate structure yields penicitrinol K (**3**) (Scheme 2). Based on this biosynthetic scheme, the absolute configurations were deduced as 3*R*, 4*S*, 2′*R*, 3′*S* - for **1**, 1*R*, 3*R*, 4*S*, 2′*R*, 3′*S* - for **2**, 1*S*, 3*R*, 4*S*, 10*S* - for **3**, 9*S* - for **4**. 

To best of our knowledge, four penicitrinones have been reported: penicitrinones A–B [4], pennicitrinone C [9] and pennicitrinone D [13] and nine penicitrinols have been reported: Penicitrinol A [4], pennicitrinol B [9], penicitrinols C–E [12] and penicitrinols F–I [14]; Three citrinolactones have been reported: citrinolactones A–C [5]. Thus, the four new citrinin derivatives-penicitrinone E (**1**), penicitrinol J (**2**), penicitrinol K (**3**) and citrinolactone D (**4**) enrich the number of penicitrinones, penicitrinols and citrinolactones respectively. Markedly, the 7-COOH of penicitrinone E (**1**) and penicitrinol J-K (**2**–**3**) were reported in both penicitrinones and penicitrinols for the first time, which complemented the Diels-Alder reaction of citrinin [10,12].

### 2.3. Cytotoxic and Antimicrobial Activity

The new compounds **1**–**4 **were tested for cytotoxic effects against the HeLa and HepG-2 cell lines using the MTT method [15]. However, they exhibited no remarkable cytotoxic activity against any of the cell lines in the concentration of 10 μg/mL (100% of cis-platinum as positive control). The results of cytotoxic tests of compounds **1**–**4** are shown in Table 4.

**Table 4:** Biological Activities of Compounds **1**–**4**.

**Compound**	**Inhibitory ratio (%)**	
	HeLa	HepG-2
**1**	-	6.3
**2**	-	25.1
**3**	-	9.2
**4**	4.0	16.1

The antimicrobial activity of compounds **1**–**4** against *Staphylococcus aureus* (CMCC26003), *Escherichia coli* (CMCC44103), *Candida albicans* (AS2.538), and *Aspergillus niger* (ACCC30005) were also evaluated by paper diffusion method with concentration of 20 μg/6 mm paper disk. Only compounds **2** and **3** showed weak antimicrobial activity against *Staphylococcus aureus* CMCC26003 with inhibition zones of 10 and 9 mm diameter, respectively (18 mm of gentamicin as positive control).

## 3. Experimental

### 3.1. General Procedures

Optical rotations were obtained on a PerkinElmer 341 automatic polarimeter. UV spectra were recorded on a Persee TU-1901 spectrophotometer. IR spectra were recorded on a Nicolet Avatar 330FT spectrometer. ^1^H-NMR, ^13^C-NMR, and DEPT spectra and 2D NMR spectra were recorded on a Bruker Avance Ⅲ-600 NMR spectrometer using TMS as internal standard, and chemical shifts were recorded as values. HRESIMS data were measured on a Bruker FT-ICR-MS mass spectrometer. ESIMS was measured on a Finnigan mass spectrometer. TLC was carried out using glass-precoated silica gel GF_254_ (Qingdao Marine Chemical, Inc., Qingdao, China) and visualized under UV light or by spraying with vanillin (contains H_2_SO_4_) ethanol reagent. Sephadex LH-20 (40–70 μm, Amersham Pharmacia Biotech AB, Uppsala, Sweden), silica gel (200–300mesh, Qingdao Marine Chemical, Inc., Qingdao, China), and Lichroprep reversed-phase RP-18 silica gel (40–63 μm, Merck, Darmstadt, Germany) were used for column chromatography (CC).

### 3.2. Fungal Material

The fungal strain *Penicillium* sp. ML226 was isolated from the sediment of Fu Gong mangrove region, Long Hai, Taiwan Strait, China. It was identified according to its morphological characteristics and ITS sequence. It was identified as a sporulating fungus by traditional morphology. A BLAST search result showed that the internal transcribed spaces (ITS) sequence of ML226 was highly homologous (96% percent similarity) to that of a *Penicillium* species (JX192960), indicating that ML226 belongs to this genus. The voucher specimen is deposited in our laboratory at−80 °C. The producing strain was prepared on potato dextrose agar slants and stored at 4 °C. 

### 3.3. Fermentation and Extraction

The fungus *Penicillium* sp. ML226 was inoculated on slope of YMG media (glucose 4.0, malt extract 10.0, yeast extract 4.0, pH 7.2) in a 250 mL solanad type flask containing solid media (25 mL/flask) at 28 °C for 4 days to afford spores. Then the spores were obtained by scraping and agitating from the slope of YMG media using 120 mL ddH_2_O. Solid media fermentation was performed with YMG media (12 L) at 28 °C for 7 days, and the spores was inoculated with inoculating loop. The cultured agar was chopped, diced and extracted with EtOAc-MeOH-AcOH (80:15:5, 3.5 liters) at room temperature overnight. The organic solution was collected through filtration, and the remaining agar residue was extracted several times more as described above until the filtrate was colourless. The combined filtrates were concentrated under vacuum to remove organic solvents. The aqueous solution was extracted five times with EtOAc to give an EtOAc solution, which was concentrated under vacuum to give a crude EtOAc extract. Then the EtOAc extract was dissolved with MeOH to give a MeOH solution. The MeOH solution was concentrated under vacuum to give a crude extract (5.00 g). 

### 3.4. Purification

The crude MeOH extract (5.00 g) was subjected to MPLC over RP-18 silica gel (170 g) using a stepwise gradient of 30, 50, 70 and 100% (v/v) MeOH in H_2_O. Then we achieved Fr.B (244 mg) and Fr.C (251 mg) obtained from 30% MeOH and Fr.D (560 mg) and Fr.E (362 mg) obtained from 50% MeOH. These fractions were further purified by repeated column chromatography (CC) on Sephadex LH-20 and silica gel.

Fr.B (244 mg) was fractionated by Sephadex LH-20 CC (140 g, eluted with MeOH) to provide five fractions (Ba–Be). Fr.Bb (50.9 mg) was fractionated by Sephadex LH-20 CC (80 g, eluted with acetone) to provide four fractions (Bb1–Bb4). Fr.Bb2 (8.0 mg) was further purified by silica gel CC [step gradient, 0–3% EtOAc in petroleum ether (PE)] to yield **9** (3.4 mg). Fr.Bb4 (3.5 mg) was further purified by silica gel CC (step gradient, 0-10% EtOAc in PE) to afford **10** (1.8 mg). 

Fr.C (251 mg) was fractionated by Sephadex LH-20 CC (140 g, eluted with MeOH) to provide seven fractions (Ca–Cg). Fr.Cb (36.6 mg) was further purified by silica gel CC (step gradient, 0–12.5% acetone in PE) to yield **1** (8.5 mg). Fr.Cd (60.0 mg) was fractionated by Sephadex LH-20 CC (80 g, eluted with acetone) to provide four fractions (Cd1–Cd4). Fr.Cd2 (10.0 mg) was further purified by silica gel CC (step gradient, 0–2.5% acetone in PE) to obtain **9** (3.2 mg). Fr.Cg (34, 2 mg) was further purified by silica gel CC (step gradient, 0-1.5% EtOAc in PE with 0.5% HCOOH) to produce **2** (4.0 mg) and **1** (2.2 mg). 

After settling, some crystals of **8** (80.7 mg) appeared in Fr.D (560 mg) and the remaining mother liquor solution (463 mg) was fractionated by Sephadex LH-20 CC (140 g, eluted with MeOH) to provide seven fractions (Da–Dg). Fr.Dc (73.6 mg) was fractionated by Sephadex LH-20 CC [80 g, eluated with MeOH/acetone (1:4)] to provide four fractions (Dc1–Dc4). Fr.Dc3 (26.0 mg) was further purified by silica gel CC (step gradient, 0–50% EtOAc in PE) to yield **6** (3.7 mg). 

Fr.E (362 mg) was fractionated by Sephadex LH-20 CC (140g, eluted with MeOH) to provide five fractions (Ec–Ee). Fr.Ec (55.1 mg) was fractionated by Sephadex LH-20 CC [80 g, eluted with MeOH/ acetone (1:4)] to provide three fractions (Ec1–Ec3). Fr.Ec2 (24.0 mg) was further purified by silica gel CC (step gradient, 0–25% acetone in PE) to yield **5** (3.0 mg). Fr.Ee (121.9 mg) was fractionated by Sephadex LH-20 CC (140g, eluted with acetone) to provide four fractions (Ee1–Ee4). Fr.Ee2 (12.9 mg) was further purified by silica gel CC (step gradient, 0–2.5% acetone in PE) to yield **3** (3.2 mg). Fr.Ee3 (43.0 mg) was further purified by silica gel CC (step gradient, 0-3.3% acetone in PE) to yield **4** (7.1 mg). Fr.Ee4 (73.0 mg) was fractionated by Sephadex LH-20 CC [80 g, eluted with MeOH/ acetone (1:4)] to provide three fractions ((Ee41–Ee43). Fr.Ee43 (36.9 mg) was further purified by silica gel CC (step gradient, 0-3.3% acetone in PE) to obtain **7** (2.6 mg).

### 3.5. Biological Assays

Cancer cell lines were derived from the cell bank of the Chinese Academy of Sciences. The cytotoxicities of the compounds **1**–**5 **were measured by the MTT (Sigma) assay [15]. The cells in 100 μL of culture medium were plated in each well of 96-well plates (Falcon, CA). After 24 h of incubation for a density of 5 × 10^3^/100 μL medium, the cells were treated in triplicate with the concentration of 10 μg/mL of every compound for 72 h at 37 °C. A 20 μL aliquot of MTT solution (5 mg/mL) was added directly to all wells and incubated for 4 h at 37 °C. To quench the reaction, 100 μL of triplex solution (10% SDS, 5% isobutanol, 12 mM HCl) was added to each well and incubated overnight at 37 °C. The optical density of each well was measured with a microplate reader (M-3350, Bio-Rad) at 595nm (excitation). Growth inhibition rates were calculated with the following equation:

$$\text{Inhibition rate }=\;\frac{{\mathrm{OD}}_{\mathrm{control}}-{\mathrm{OD}}_{\mathrm{treated}}}{{\mathrm{OD}}_{\mathrm{control}}-{\mathrm{OD}}_{\mathrm{blank}}}\times 100\%$$

### 3.6. Spectral Data

*Penicitrinone E*(**1**): red, amorphous powder; ${\left[\alpha \right]}_{D}^{25}$ 0 (0.002, MeOH); UV (MeOH) λ_max_ (log ε) 204 (2.74), 214 (2.76), 232 (2.58), 258 (2.37), 278 (2.49), 315 (2.34), 400 (2.49) nm; IR (KBr) ν_max_ 3453, 2967, 2928, 2873, 2358, 2332, 1687, 1641, 1611, 1527, 1506, 1451, 1408, 1380, 1325, 1269, 1154, 1101, 1060, 1026, 993, 933, 899, 858, 822, 748, 700, 675, 652, 552, 505 cm^−1^; *R*_f_ = 0.318 (PE : acetone = 1:1), *R*_f_ = 0.611 (PE : acetone = 1:1, with formic acid); ^1^H- and ^13^C-NMR (see Table 1); HRESIMS *m/z* 425.15965 [M + H]^+^ (calcd. for C_24_H_25_O_7_, 425.1600).

*Penicitrinol J*(**2**): pale yellow, amorphous powder; ${\left[\alpha \right]}_{D}^{25}$ −30.0 (0.001, MeOH); UV (MeOH) λ_max_ (log ε) 203 (3.05), 215 (3.12), 257 (2.61), 277 (2.56), 315 (2.55), 400 (2.51) nm; IR (KBr) ν_max_ 3343, 2967, 2925, 2869, 2364, 2338, 1684, 1636, 1586, 1527, 1504, 1451, 1421, 1385, 1328, 1274, 1239, 1165, 1136, 1074, 1022, 996, 974, 929, 907, 858, 815, 778, 754, 729, 704, 636, 550 cm^−1^; *R*_f_ = 0.591 (PE : acetone = 1:1), *R*_f_ = 1 (PE:acetone = 1:1, with formic acid); ^1^H- and ^13^C-NMR (see Table 1); HRESIMS *m/z* 427.17601 [M + H]^+^ (calcd. for C_24_H_27_O_7_, 427.1757).

*Penicitrinol K* (**3**): white, amorphous powder; ${\left[\alpha \right]}_{D}^{25}$ −125.2 (0.002, MeOH); UV (MeOH) λ_max_ (log ε) 213(2.68), 257 (2.34), 325 (1.90) nm; IR (KBr) ν_max_ 3433, 3230, 2970, 2930, 2872, 2360, 2334, 1687, 1623, 1589, 1445, 1419, 1386, 1353, 1298, 1270, 1232, 1200, 1169, 1105, 1079, 1025, 905, 876, 809, 753, 729, 683, 582, 549 cm^−1^; *R*_f_ = 0.524 (CHCl_3_ : MeOH = 10:1); ^1^H- and ^13^C-NMR (see Table 2); The citrinin moiety’s HRESIMS *m/z* 273.07299 [M + Na]^+^ (calcd. for C_13_H_14_O_5_ Na, 273.0739); ESIMS *m/z* 331.2 [M + Na]^+^ and 291.4 [M − H_2_O + H]^+^.

*Citrinolactone D*(**4**): white, amorphous powder; ${\left[\alpha \right]}_{D}^{25}$ +6.7 (0.004, MeOH); UV (MeOH) λ_max_ (log ε) 204 (2.35), 229 (2.45), 237 (2.49), 257 (2.31), 325 (1.80) nm; IR (KBr) ν_max_ 3437, 2965, 2924, 2824, 2365, 2337, 1653, 1622, 1599, 1490, 1451, 1364, 1296, 1253, 1206, 1141, 1083, 1031, 1011, 941, 922, 869, 822, 784, 757, 698, 656, 619, 570, 513, 471 cm^−1^; *R*_f_ = 0.675 (PE : acetone = 2:1); ^1^H- and ^13^C-NMR (see Table 3); HRESIMS *m/z* 269.07804 [M + Na]^+^ (calcd. for C_14_H_14_O_4_ Na, 269.0790).

## 4. Conclusions

Four new compounds, penicitrinone E (**1**), penicitrinol J (**2**), penicitrinol K (**3**) and citrinolactone D (**4**), were isolated together with six known compounds from the marine-derived *Penicillium* sp. ML226. Penicitrinone E (**1**), penicitrinol J (**2**) and penicitrinol K (**3**) showed modest selective cytotoxixity against HepG-2 cell line. Citrinolactone D (**4**) showed weak cytotoxixity against HepG-2 and HeLa cell lines. penicitrinol J (**2**) and penicitrinol K (**3**) showed mild antimicrobial activity against *Staphylococcus aureus*.

## Supplementary Materials

The NMR spectra and HRESIMS spectra of compounds **1**–**4** can be accessed at: http://www.mdpi.com/1420-3049/18/5/5723/s1.
