# Supplementary material for: Four New Citrinin Derivatives from a Marine-Derived Penicillium sp. Fungal Strain
Source: Molecules. 2013 May 16;18(5):5723–35. doi: 10.3390/molecules18055723 (PMC6270126; doi:10.3390/molecules18055723)

Supplementary Materials

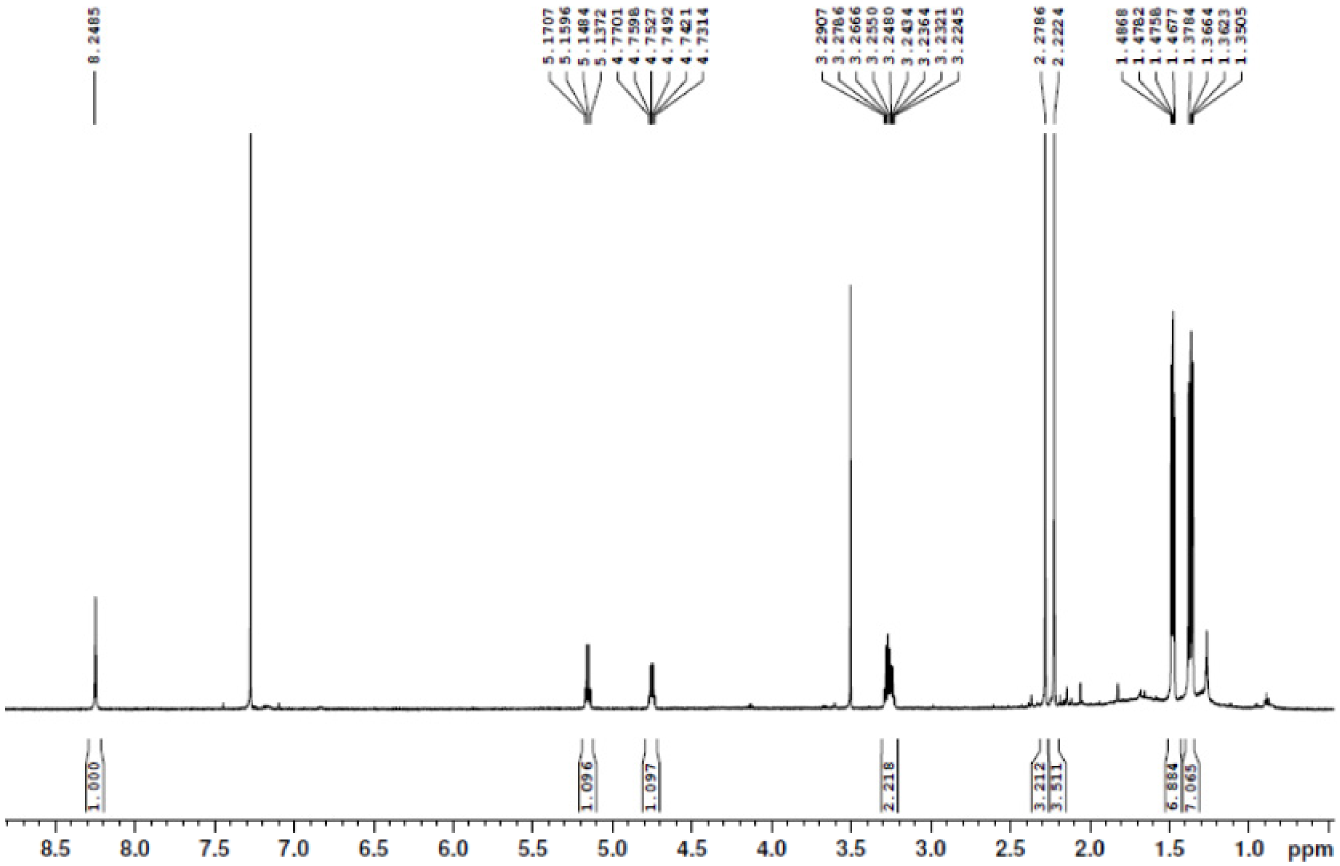

Figure S1. ^{1}H-NMR spectrum (600 MHz, $CDCl_3$) of penicitrinone E.

Figure S2. ^{13}C-NMR and DEPT spectrum (150 MHz, $CDCl_3$) of penicitrinone E.

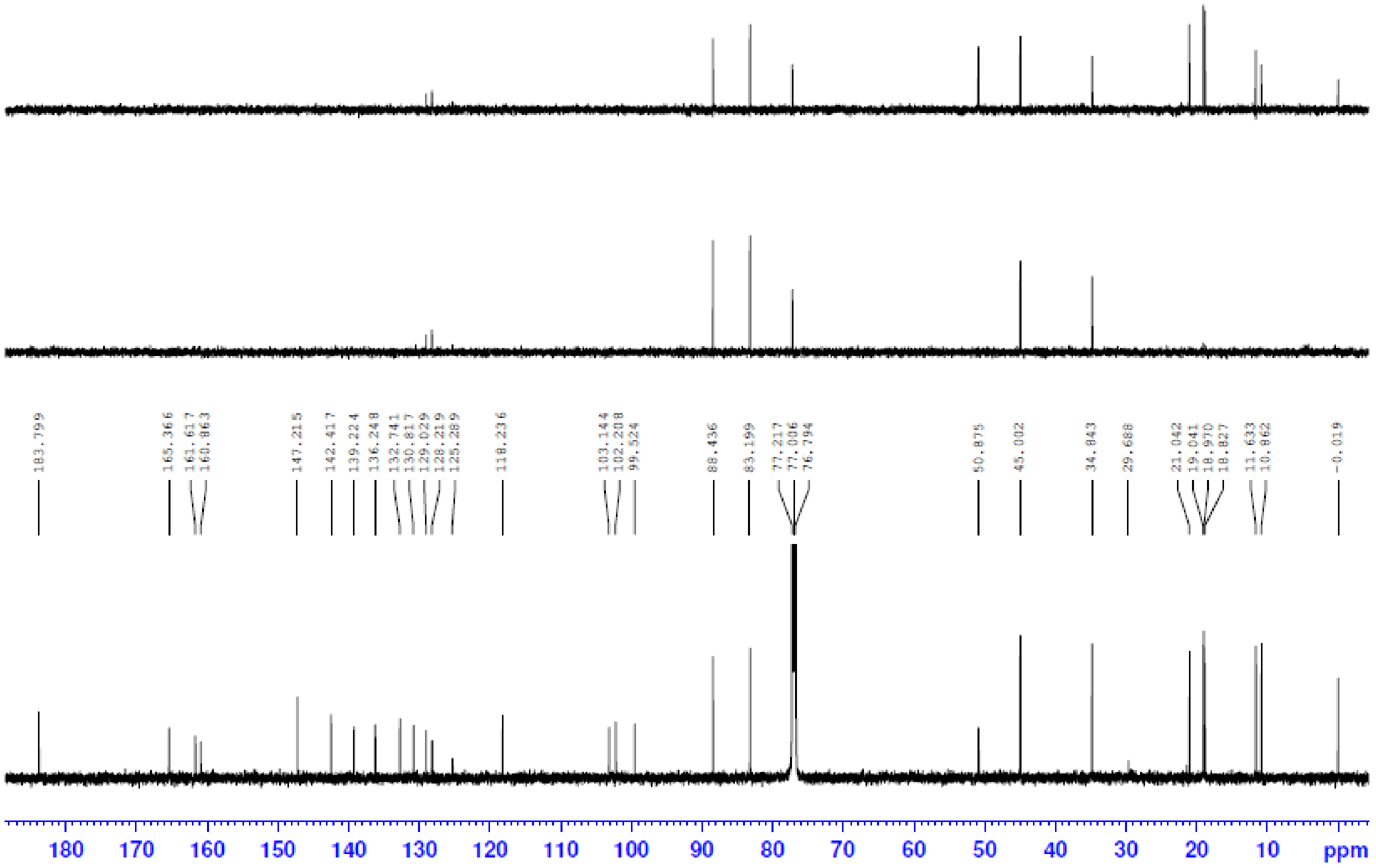

Figure S3. HMQC spectrum (600 MHz, $CDCl_3$) of penicitrinone E.

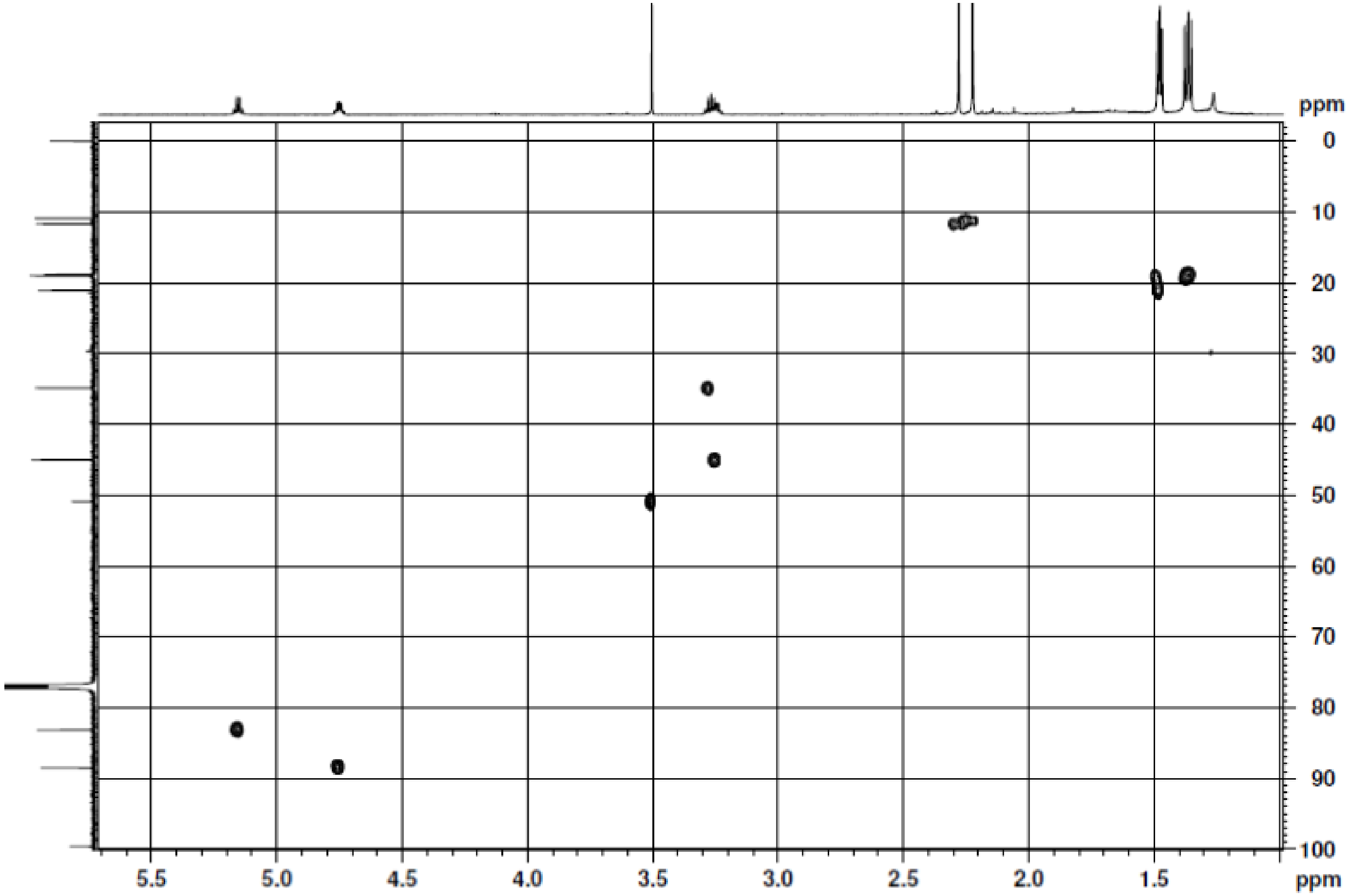

Figure S4. ^{1}H-^{1}H COSY spectrum (600 MHz, $CDCl_3$) of penicitrinone E.

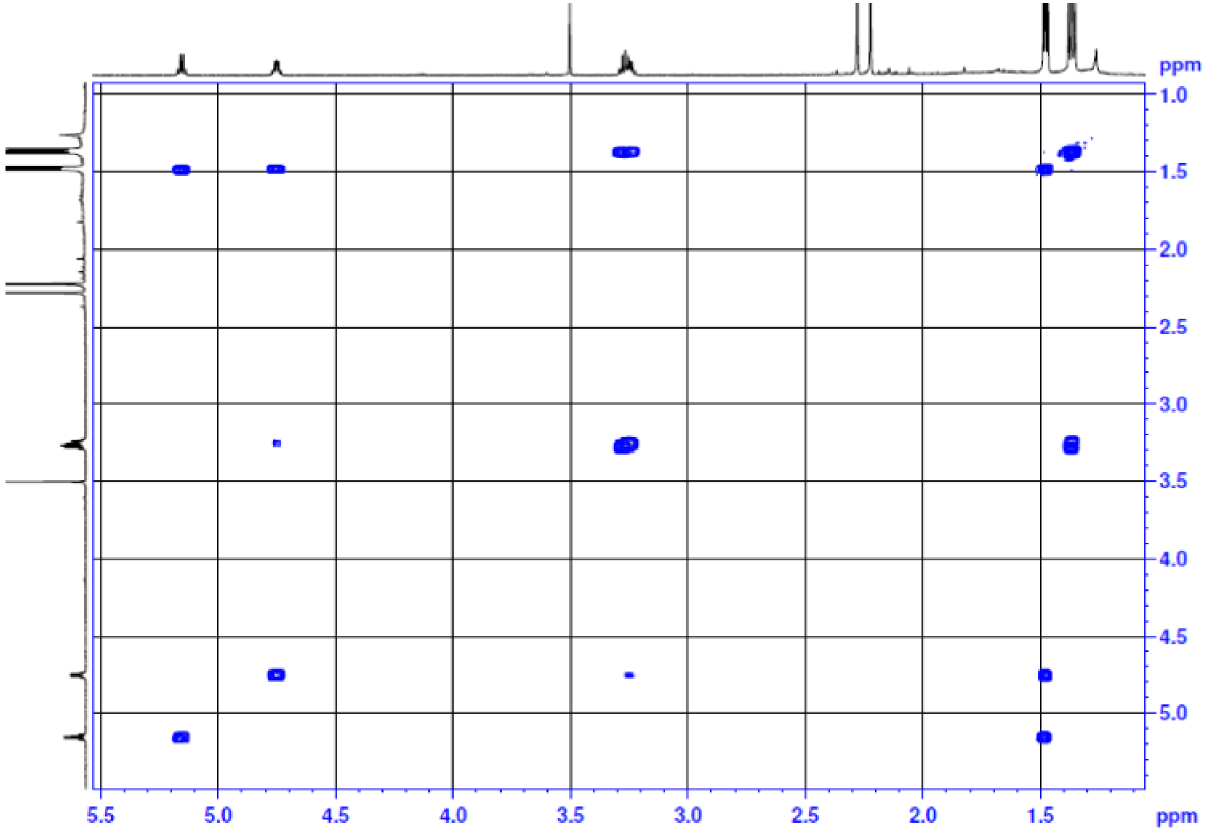

Figure S5. HMBC spectrum (600 MHz, $CDCl_3$) of penicitrinone E.

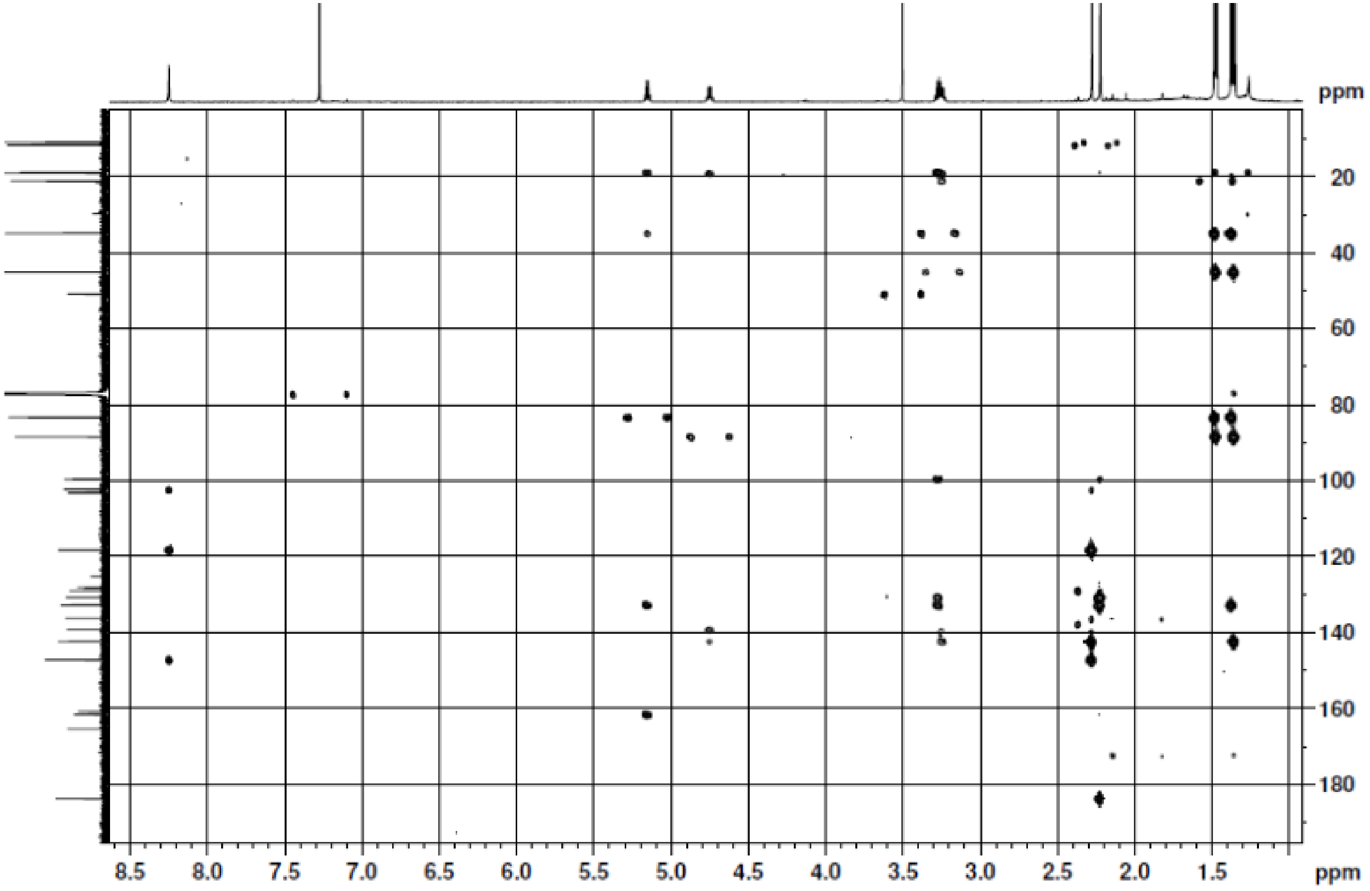

Figure S6. NOESY spectra (600 MHz, $CDCl_3$) of penicitrinone E.

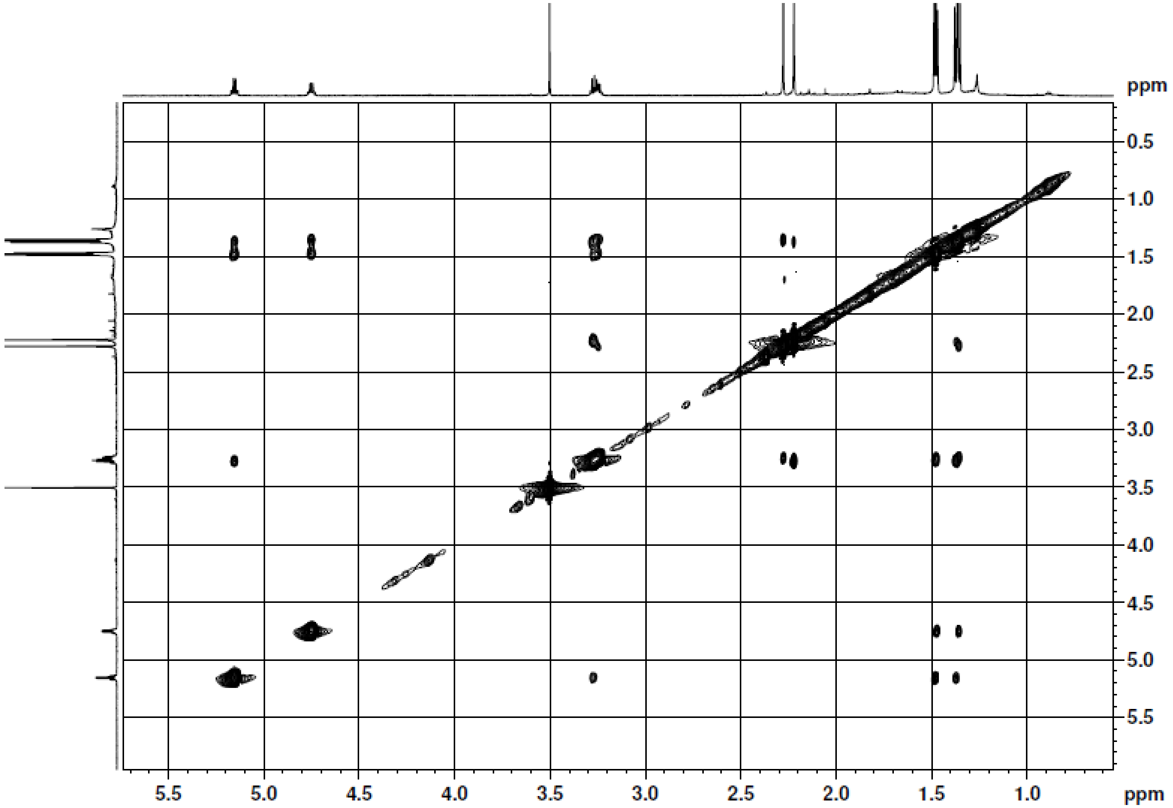

Figure S7. HRESIMS spectrum of penicitrinone E.

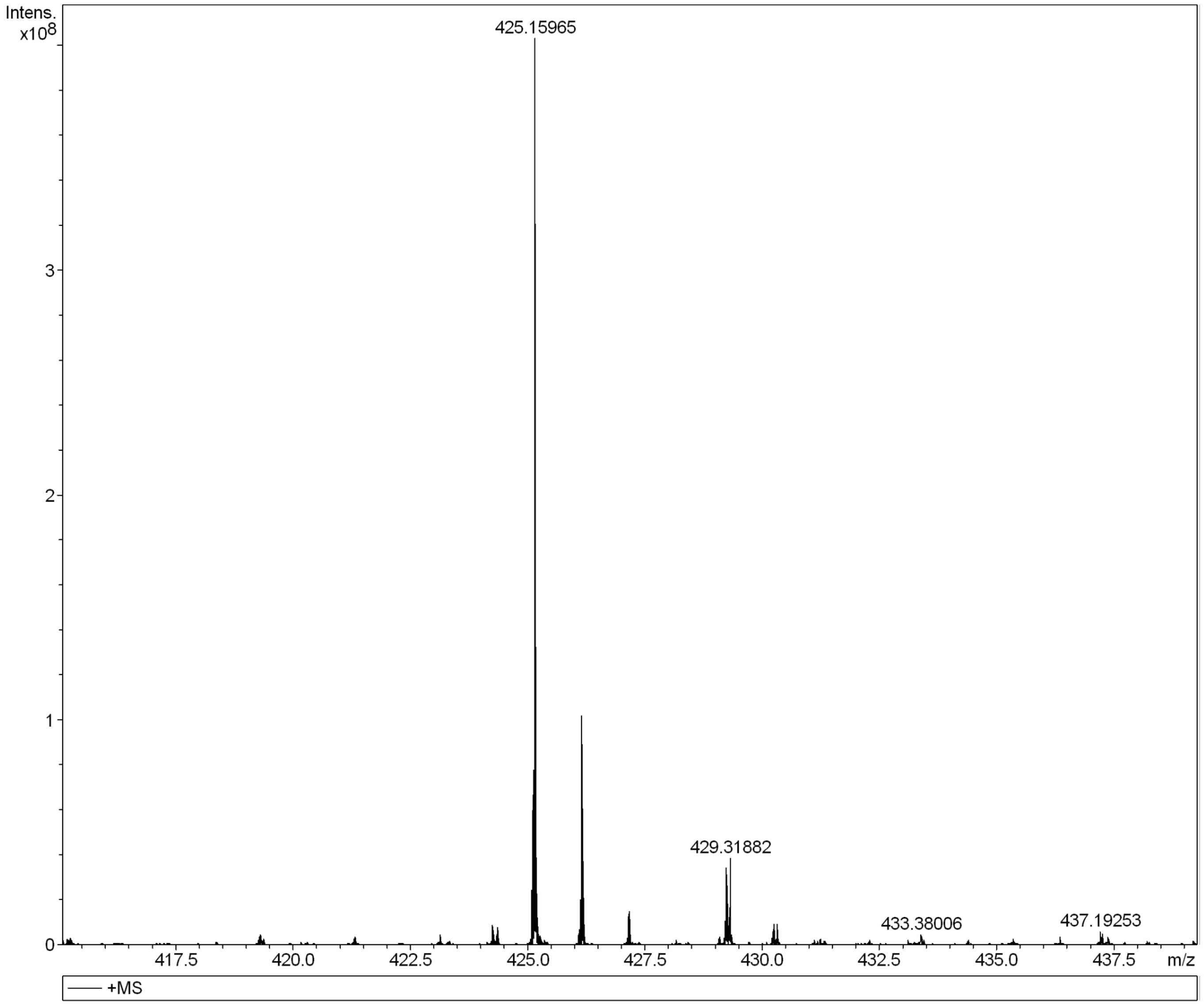

Figure S8. ^{1}H-NMR spectrum (600 MHz, $CDCl_3$) of penicitrinol J.

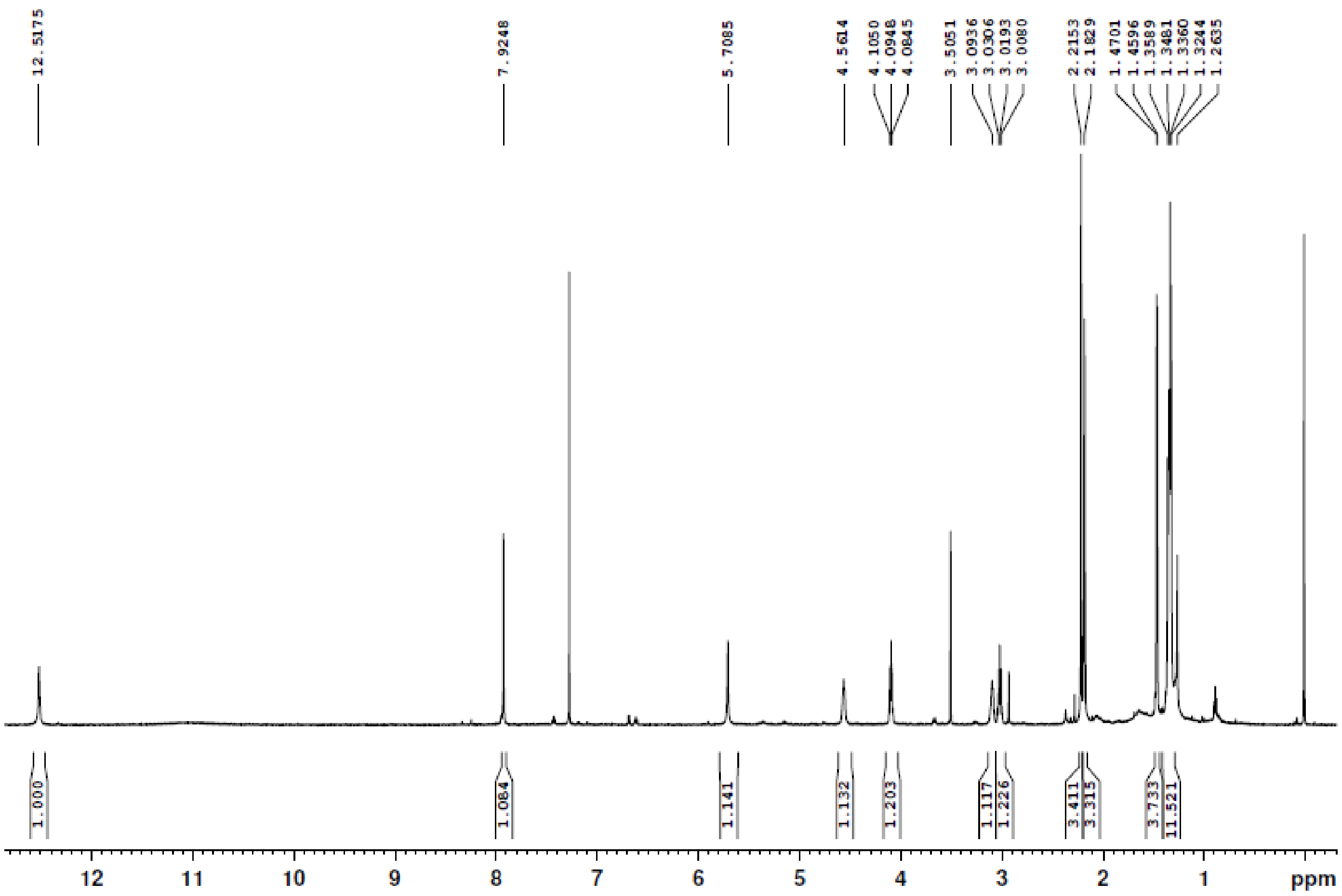

Figure S9. ^{13}C-NMR and DEPT spectra (150 MHz, $CDCl_3$) of penicitrinol J.

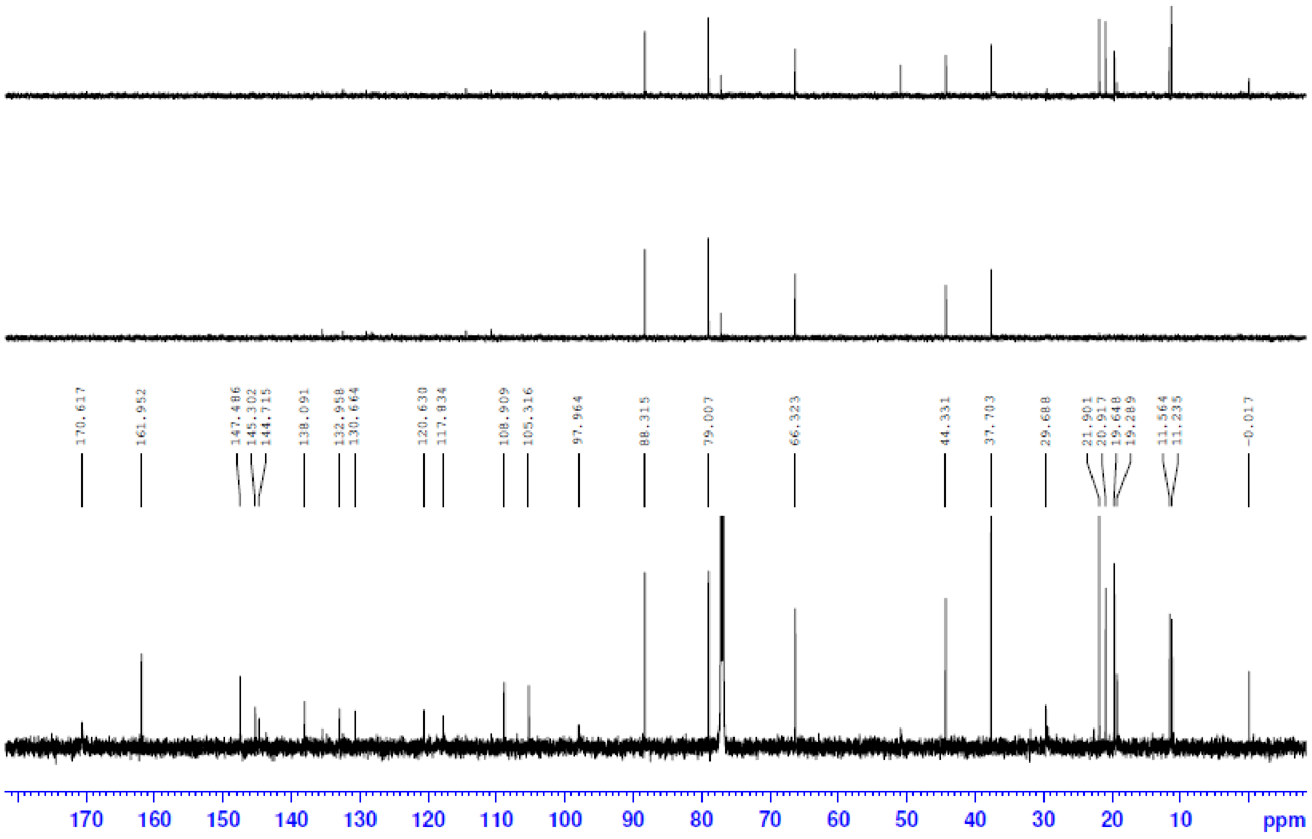

Figure S10. HMQC spectrum (600 MHz, $CDCl_3$) of penicitrinol J.

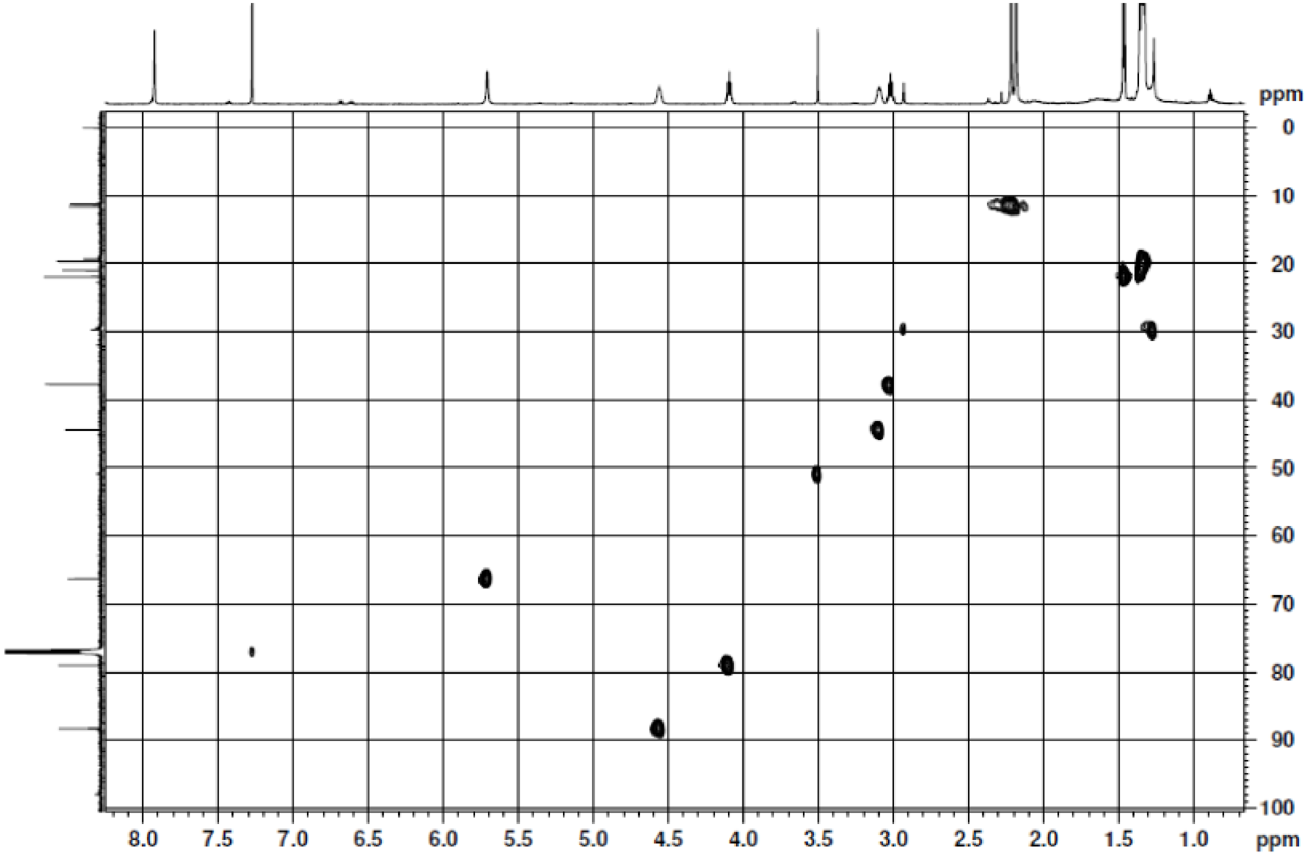

Figure S11. ^{1}H-^{1}H COSY spectrum (600 MHz, $CDCl_3$) of penicitrinol J.

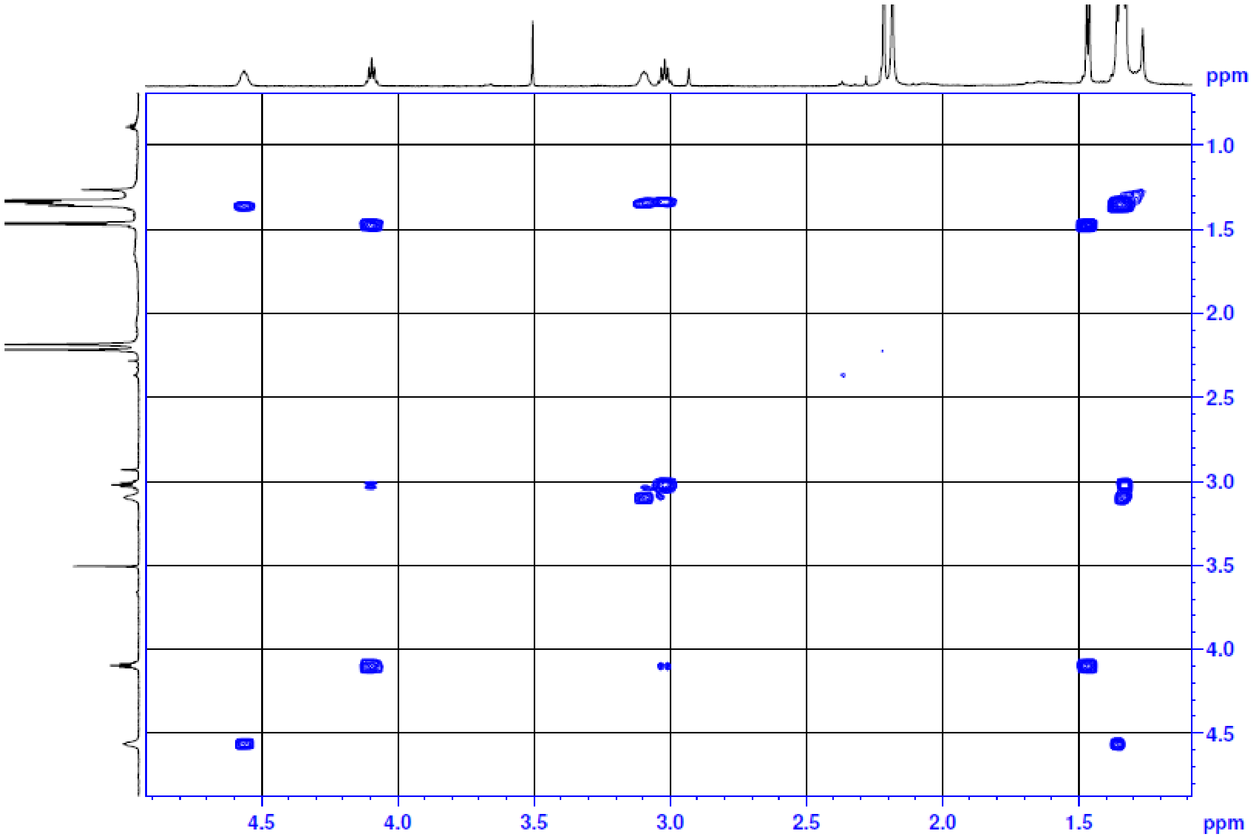

Figure S12. HMBC spectrum (600 MHz, $CDCl_3$) of penicitrinol J.

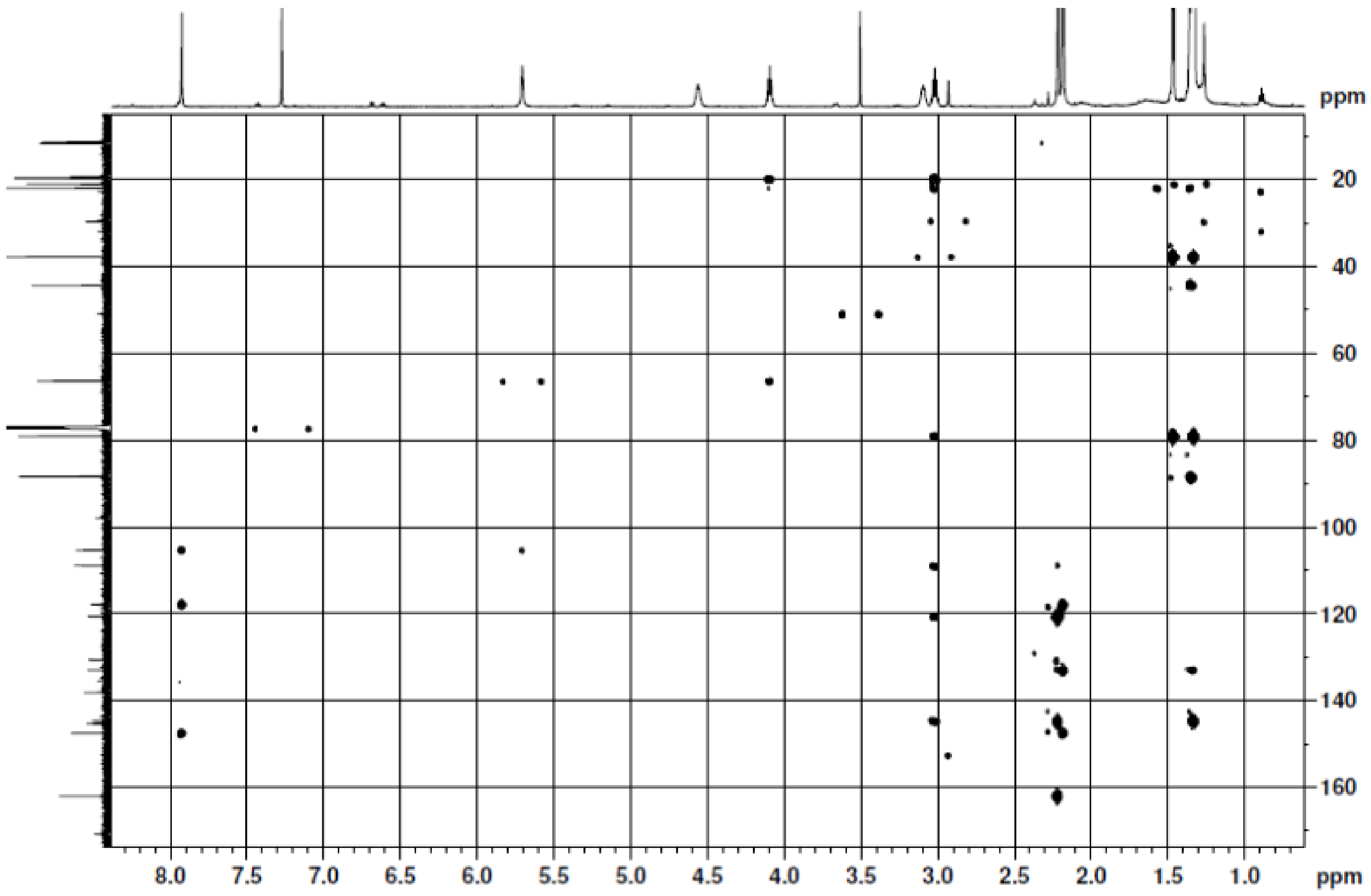

Figure S13. NOESY spectrum (600 MHz, $CDCl_3$) of penicitrinol J.

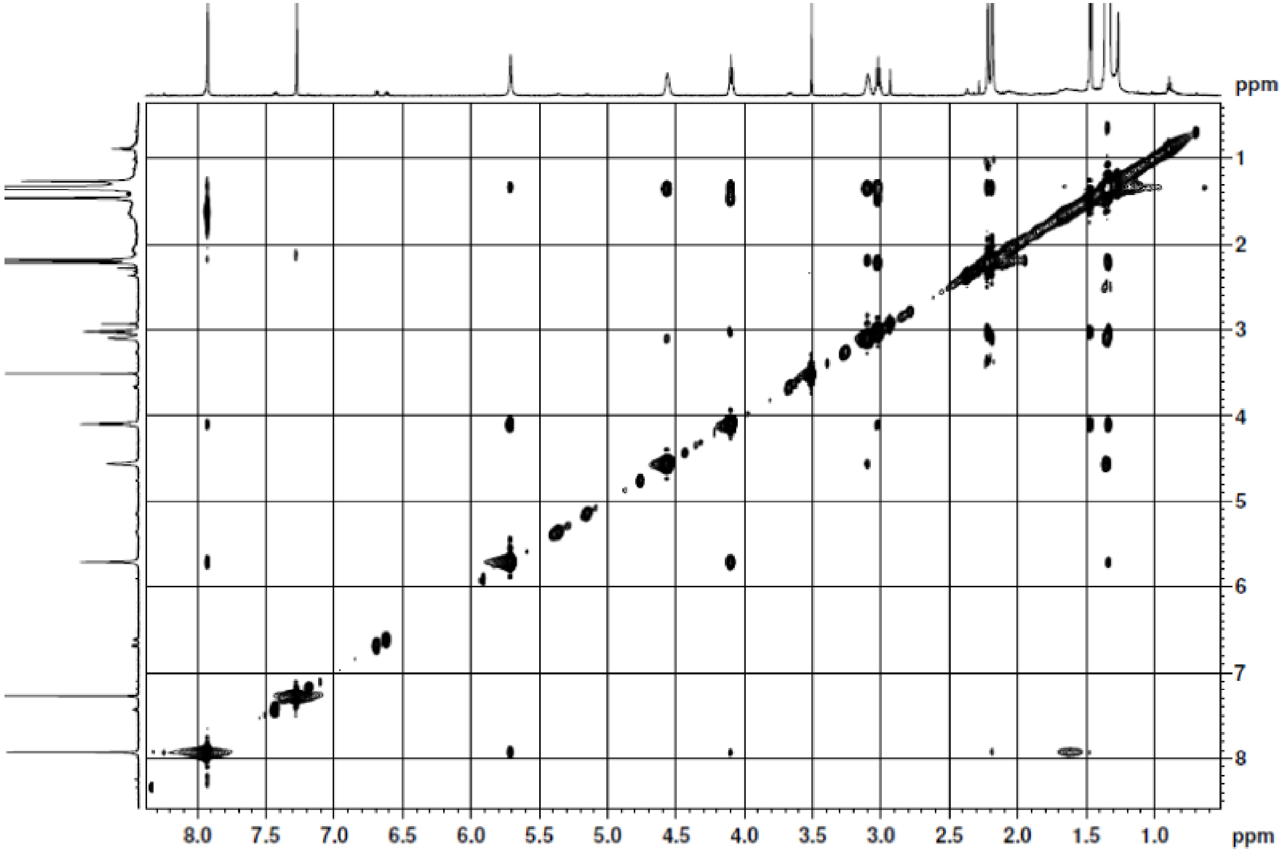

Figure S14. HRESIMS spectrum of penicitrinol J.

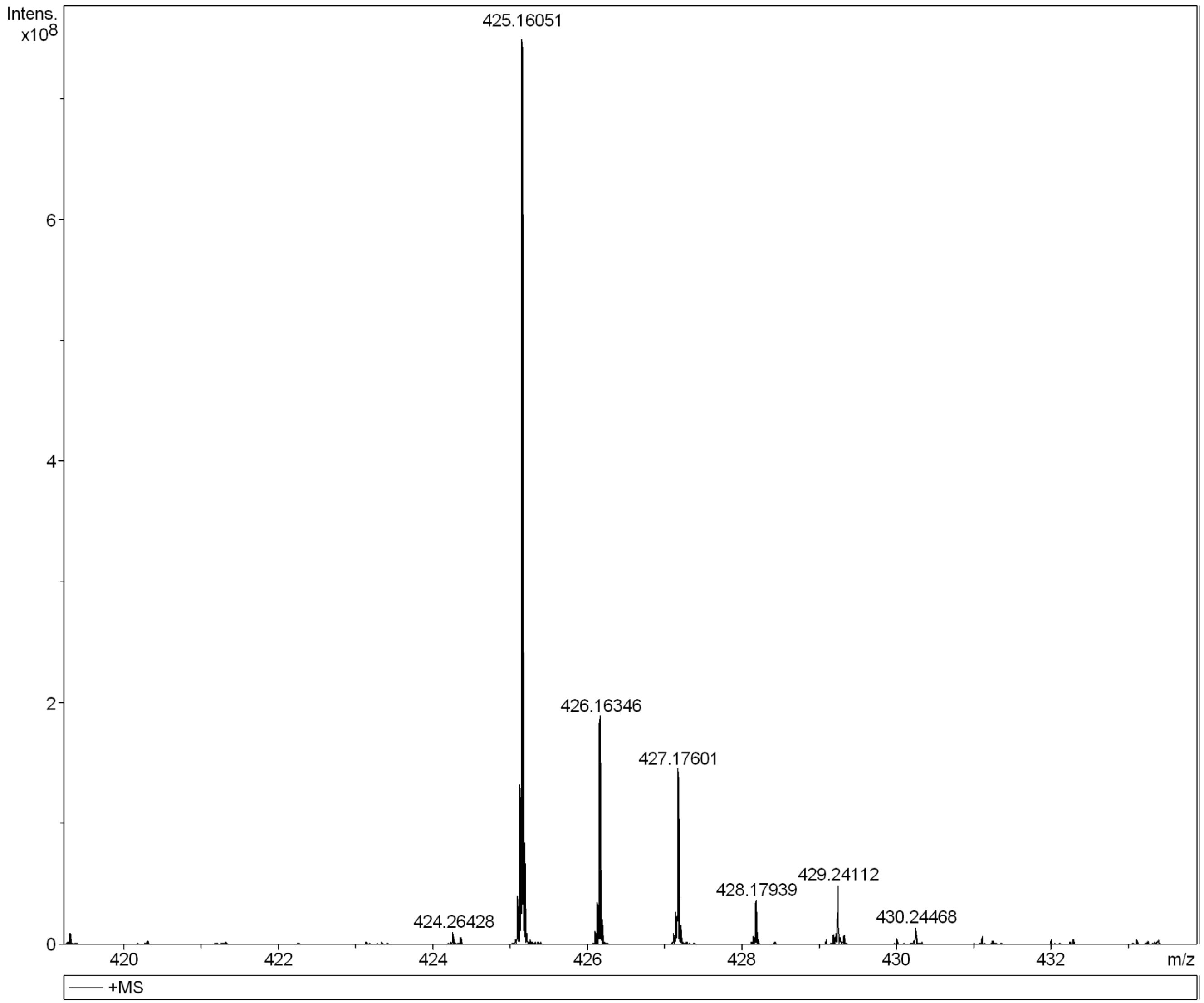

Figure S15. ^{1}H-NMR spectrum (600 MHz, $CDCl_3$) of penicitrinol k.

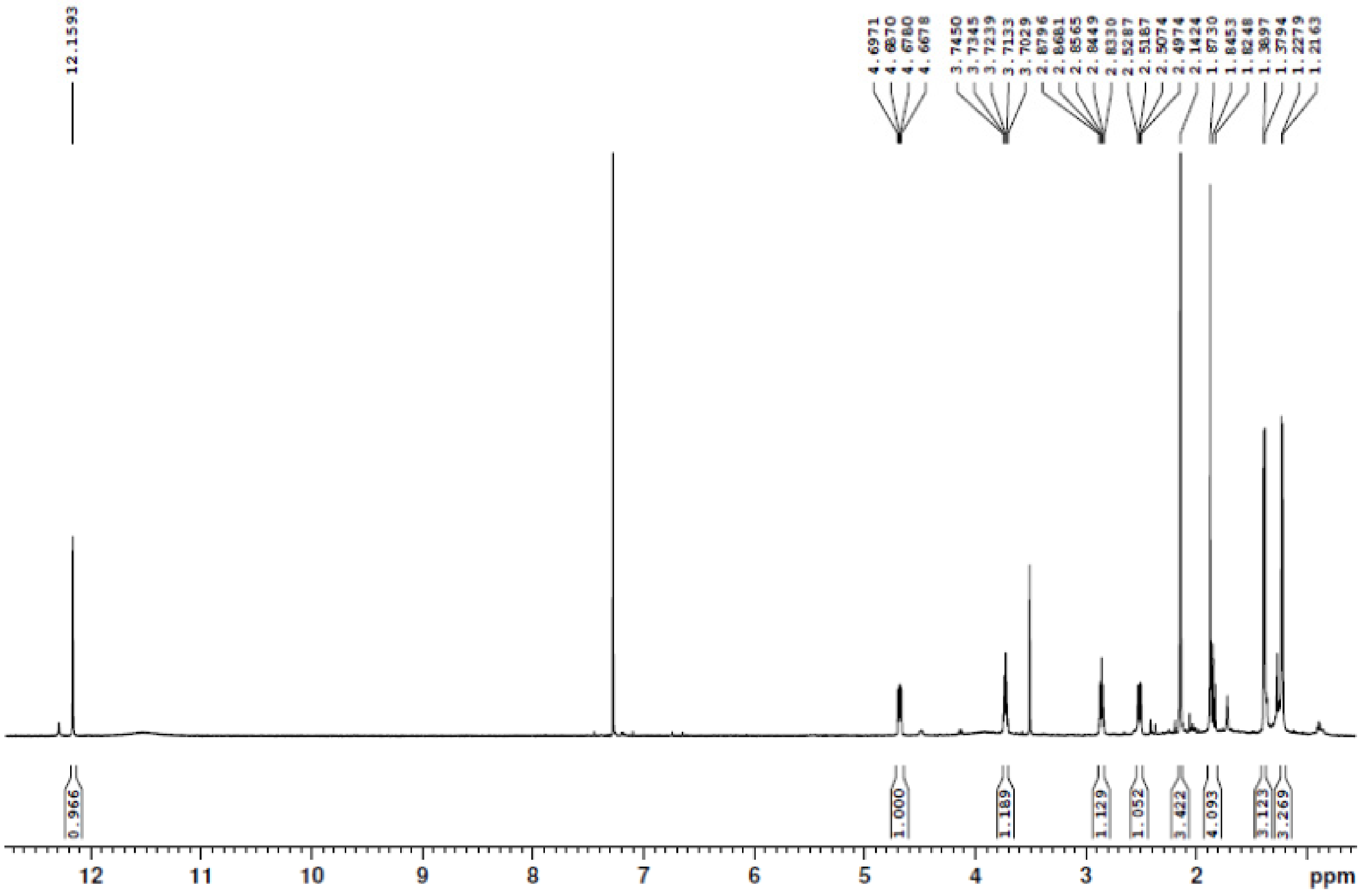

Figure S16. ^{13}C-NMR and DEPT spectra (150 MHz, $CDCl_3$) of penicitrinol k.

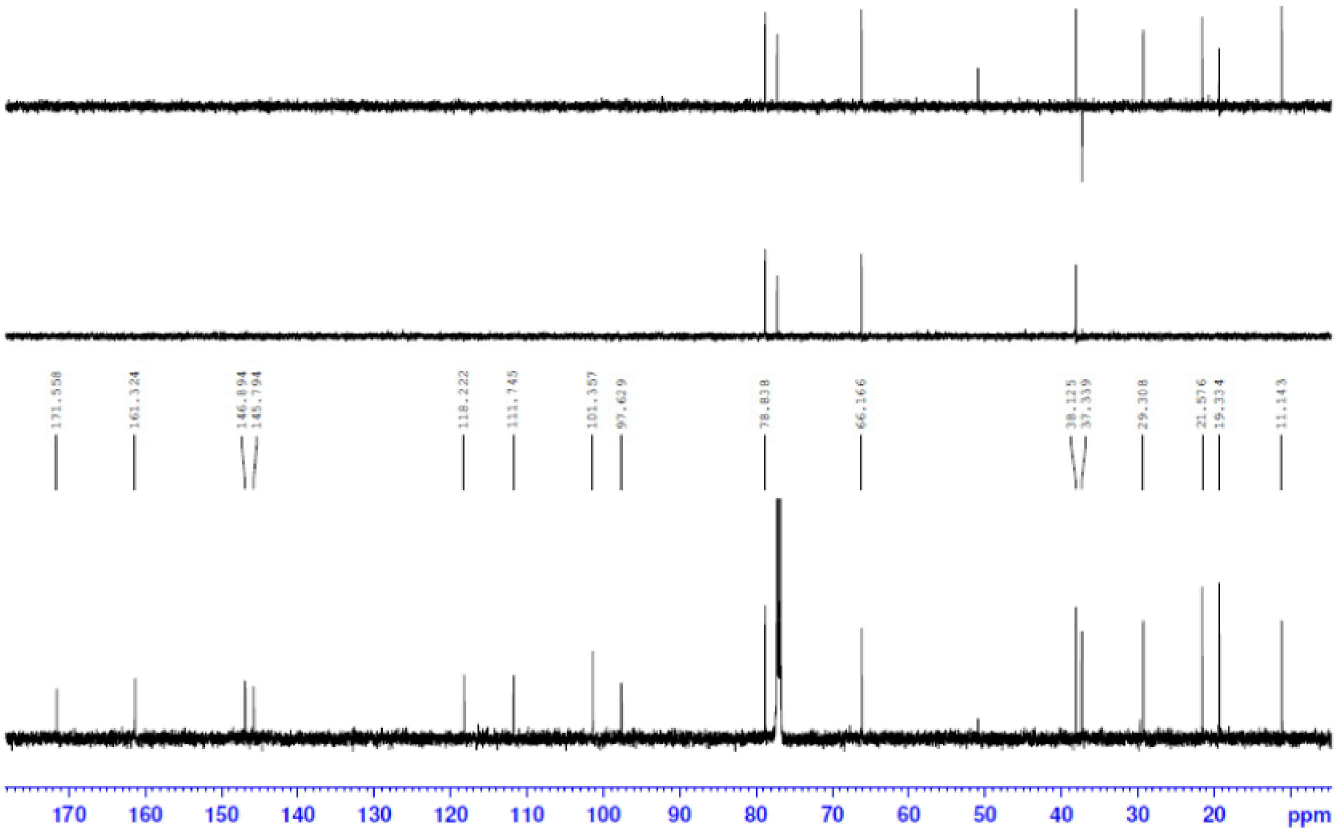

Figure S17. HMQC spectrum (600 MHz, $CDCl_3$) of penicitrinol k.

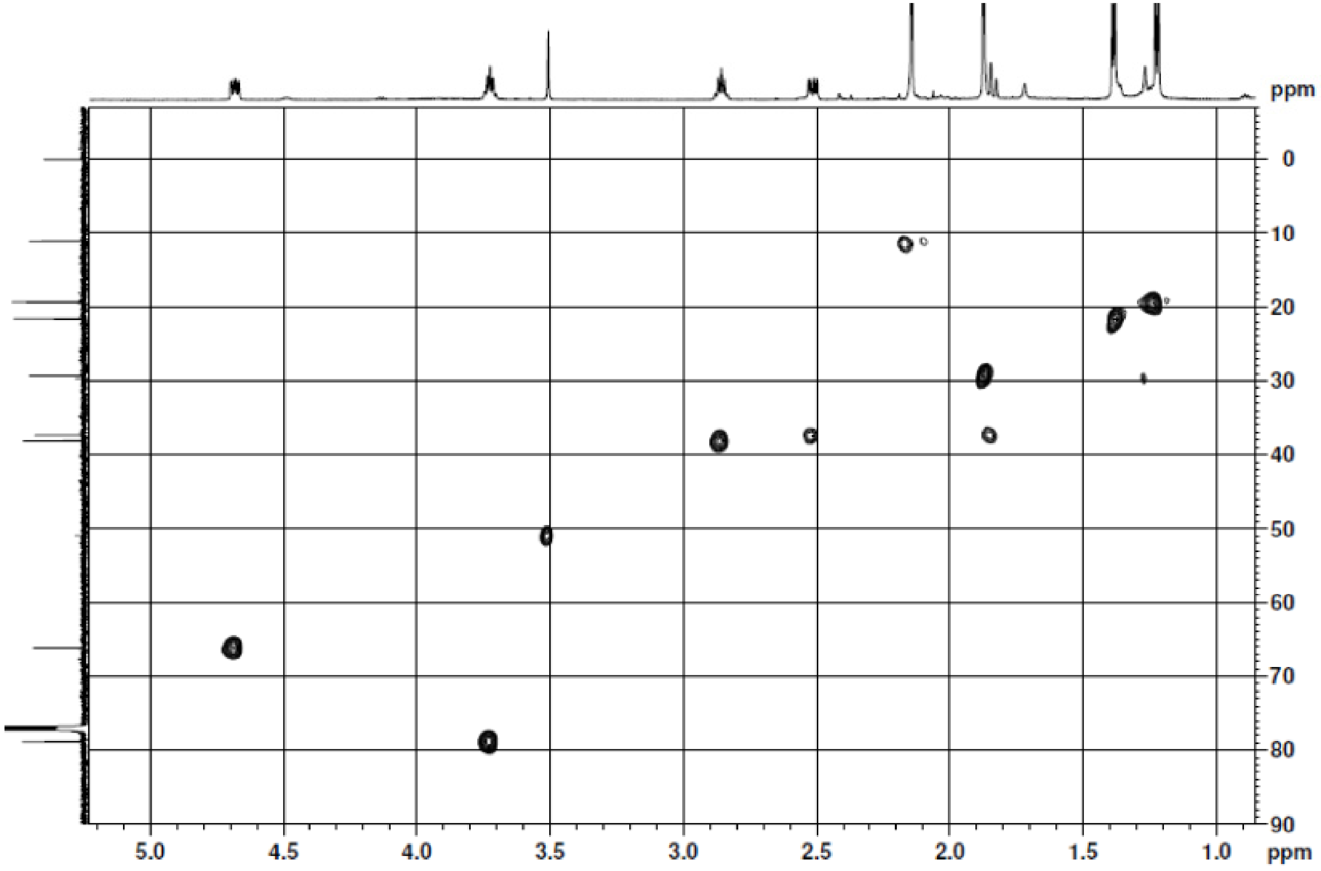

Figure S18. ^1H-^1H COSY spectrum (600 MHz, $CDCl_3$) of penicitrinol k.

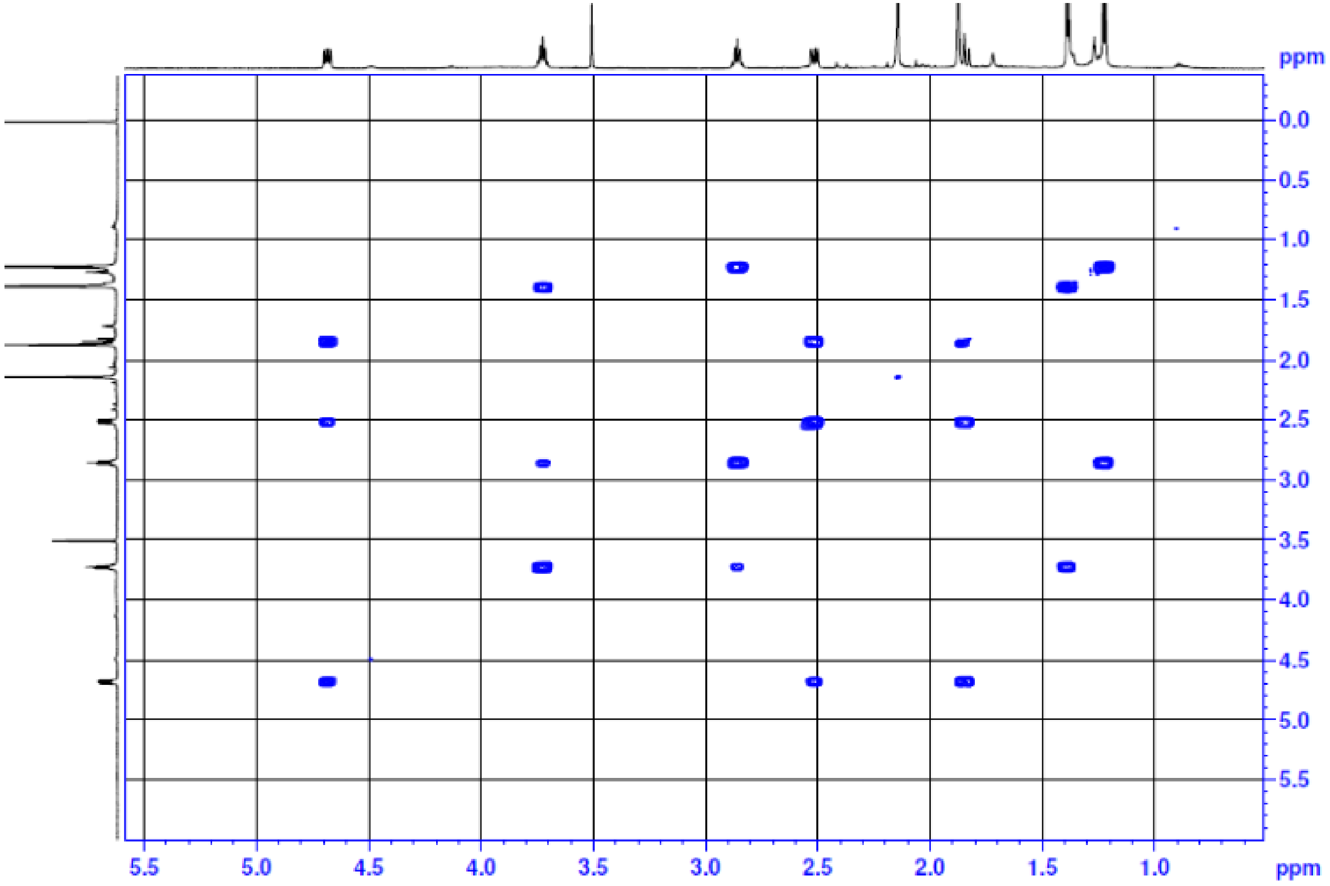

Figure S19. HMBC spectrum (600 MHz, $CDCl_3$) of penicitrinol k.

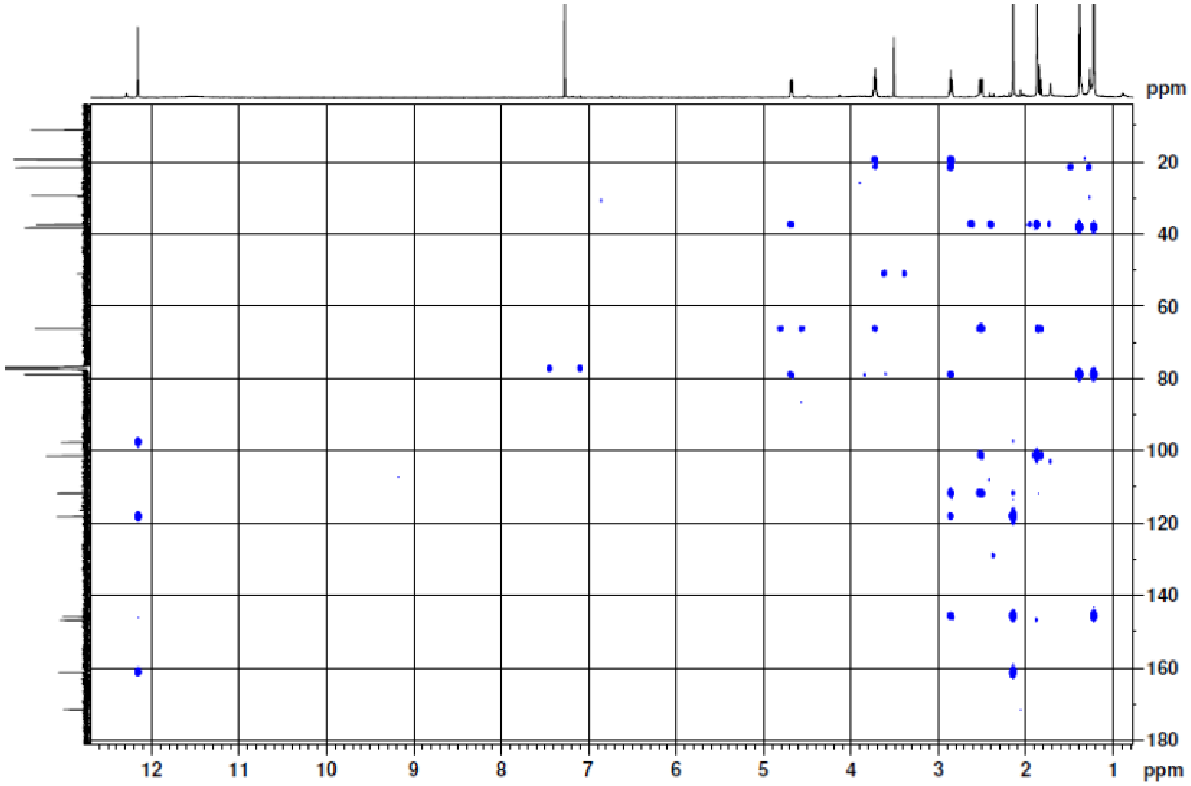

Figure S20. NOESY spectrum (600 MHz, $CDCl_3$) of penicitrinol k.

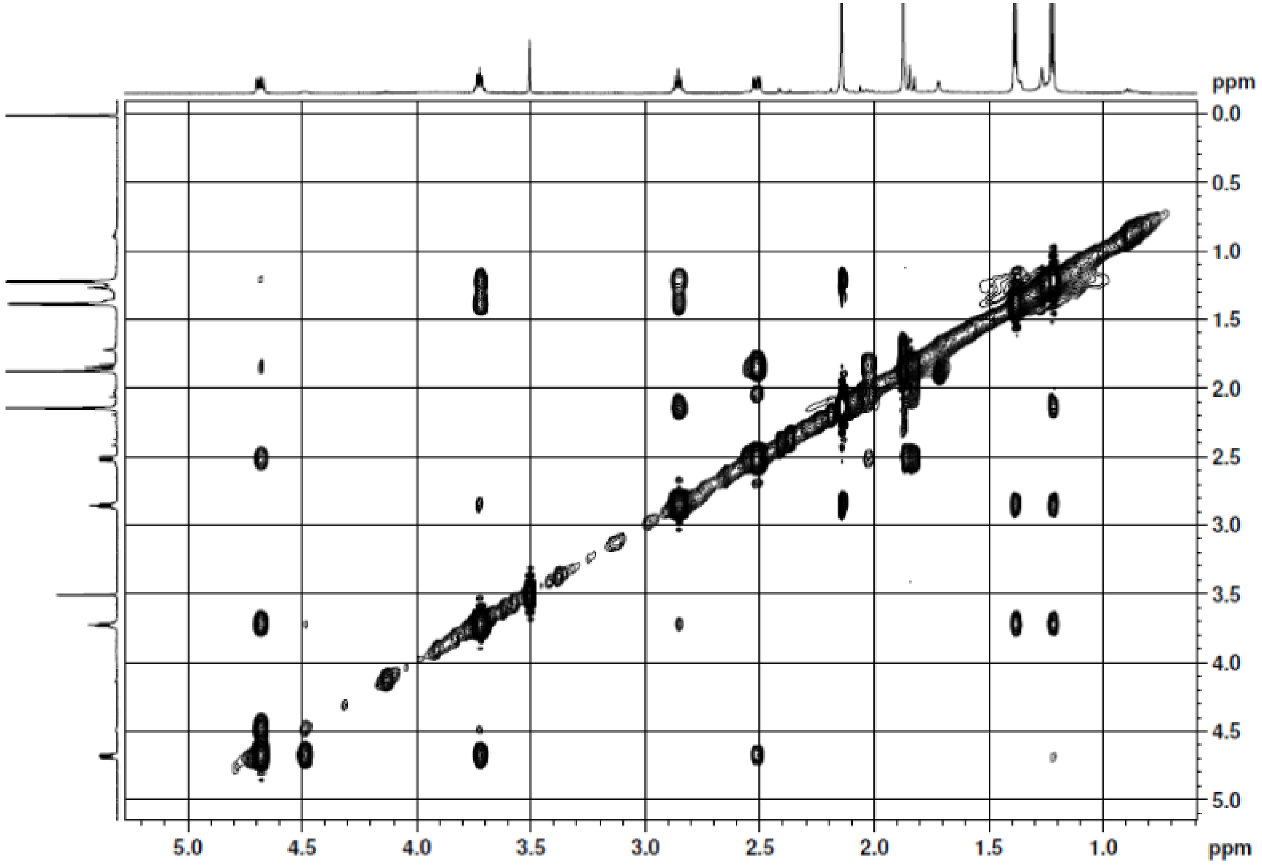

Figure S21. HRESIMS spectrum of the benzopyran moiety of penicitrinol k.

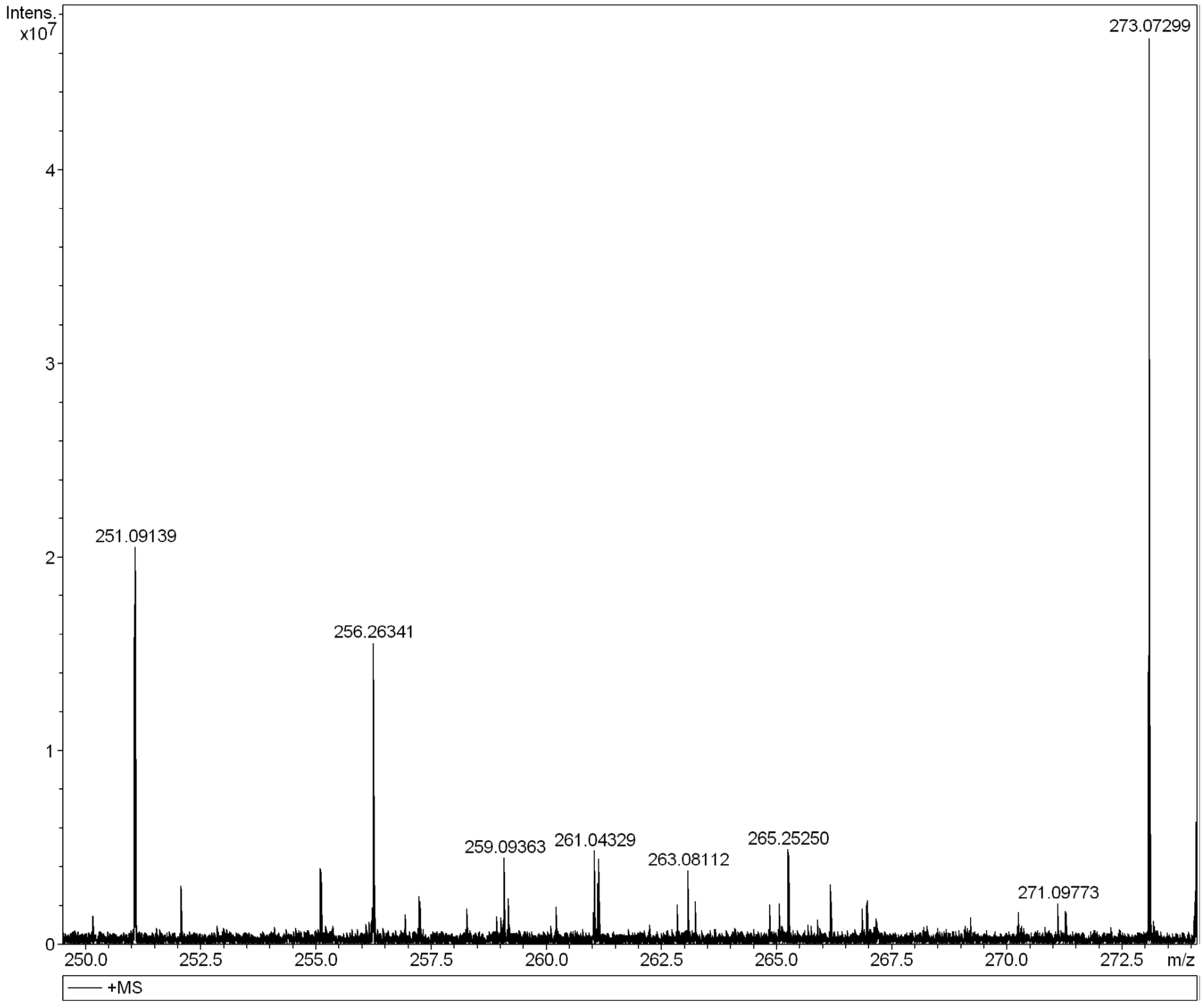

Figure S22. ^{1}H-NMR spectrum (600 MHz, $CDCl_3$) of citrinolactone D.

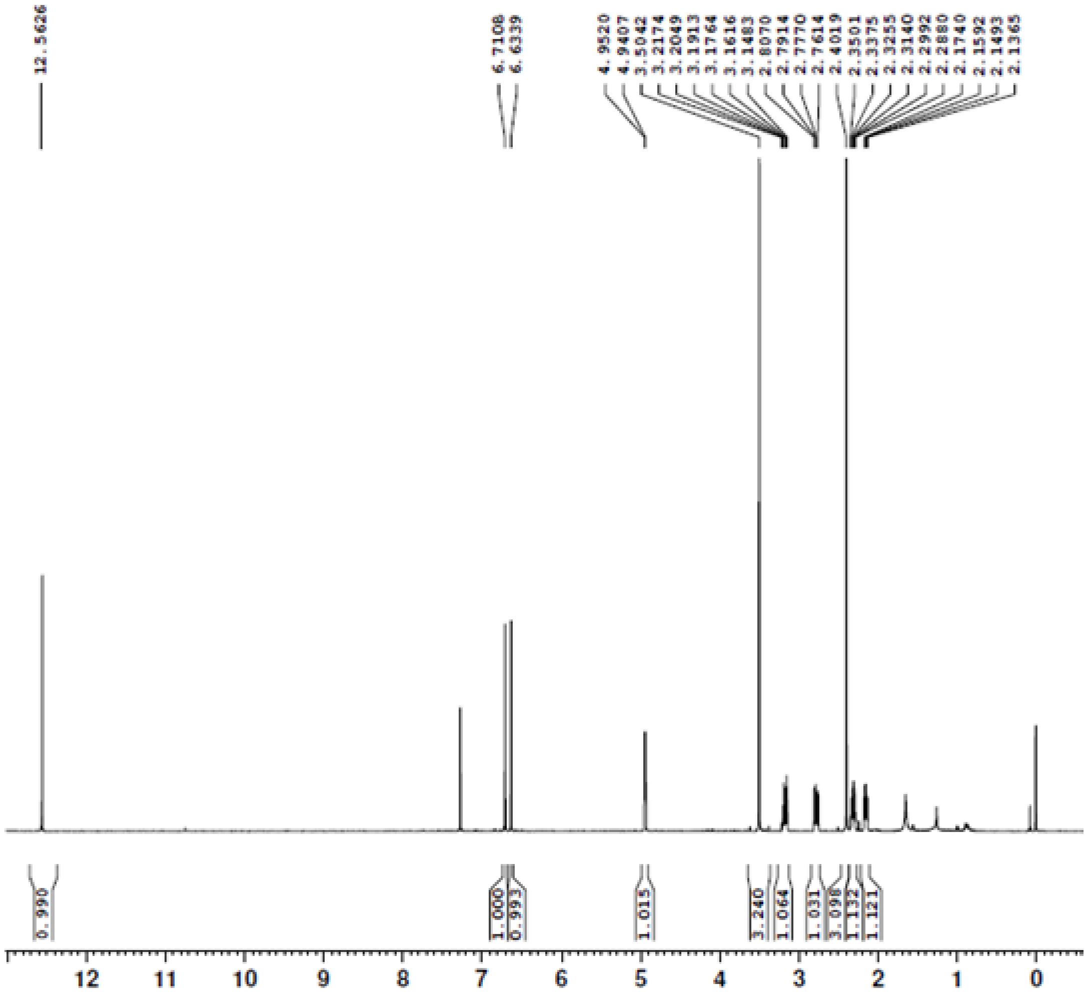

Figure S23. ^{13}C-NMR spectrum (150 MHz, $CDCl_3$) of citrinolactone D.

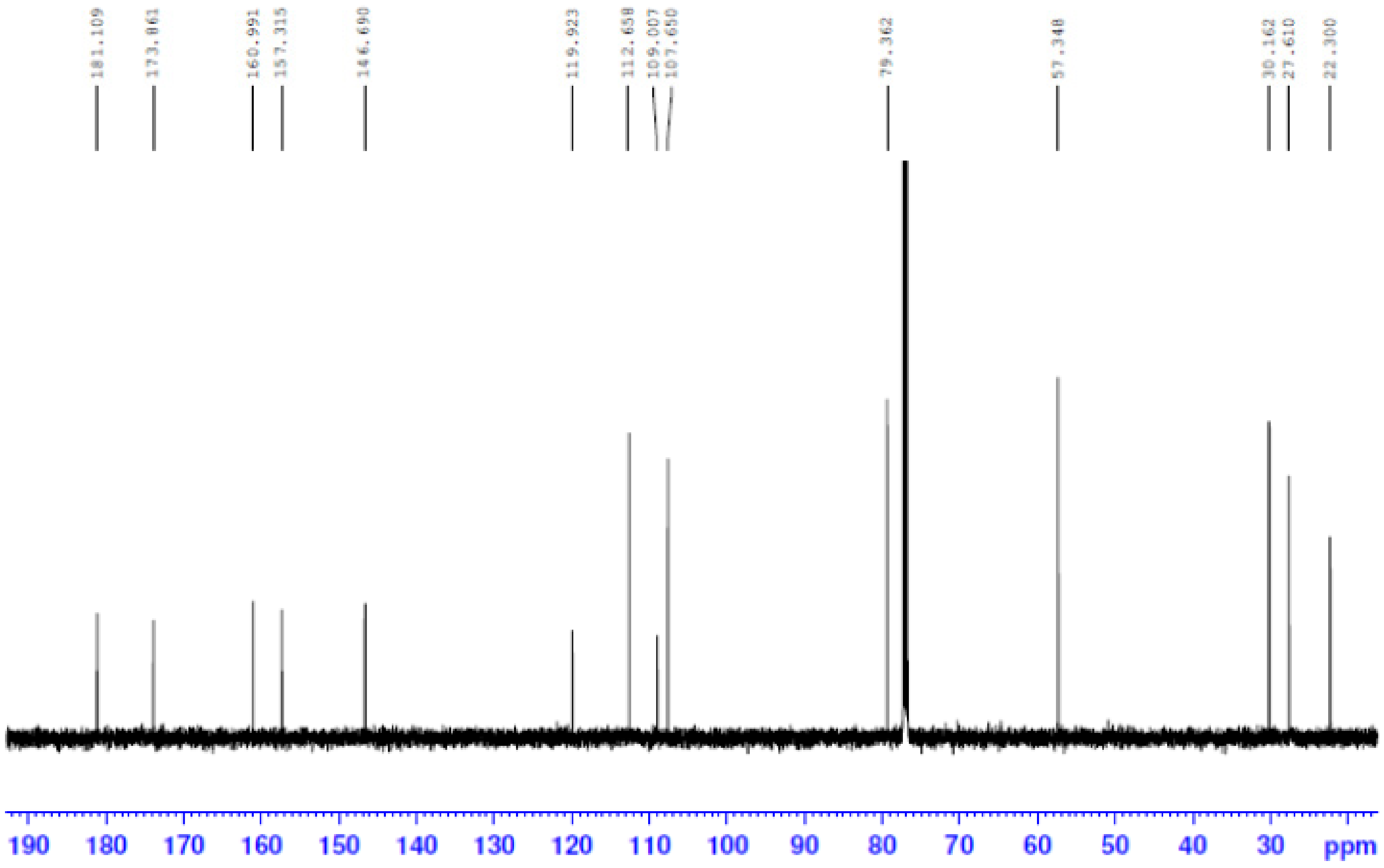

Figure S24. HMQC spectrum (600 MHz, $CDCl_3$) of citrinolactone D.

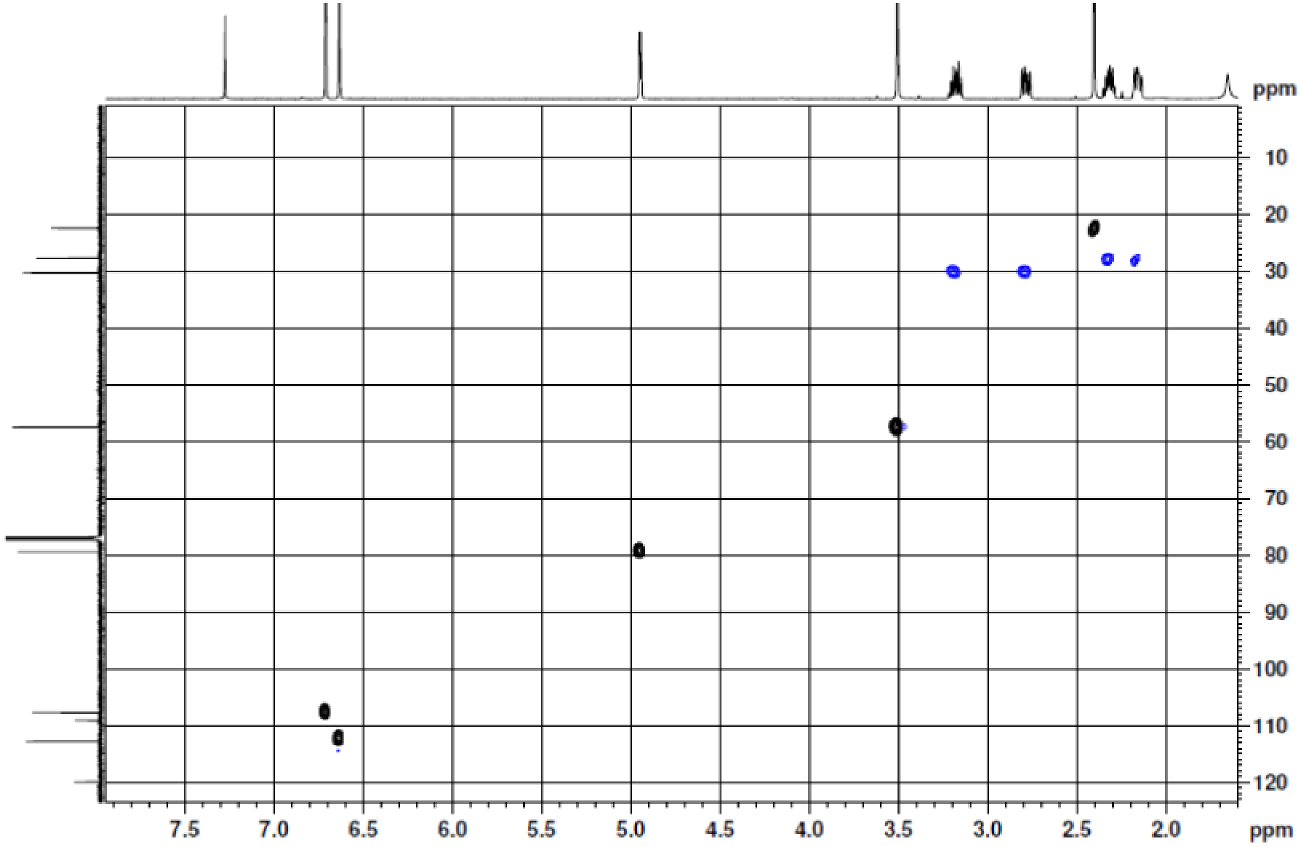

Figure S25. ^{1}H-^{1}H COSY spectrum (600 MHz, $CDCl_3$) of citrinolactone D.

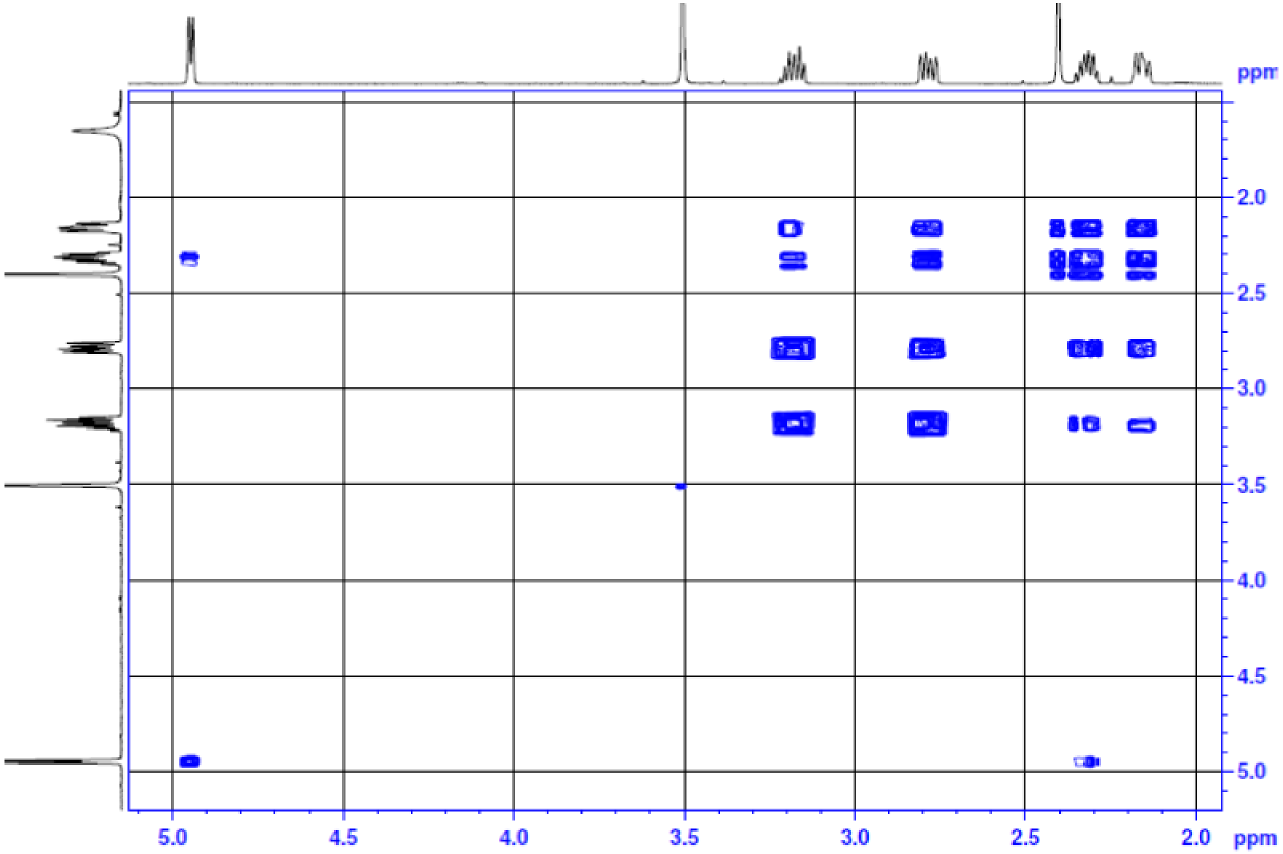

Figure S26. HMBC spectrum (600 MHz, $CDCl_3$) of citrinolactone D.

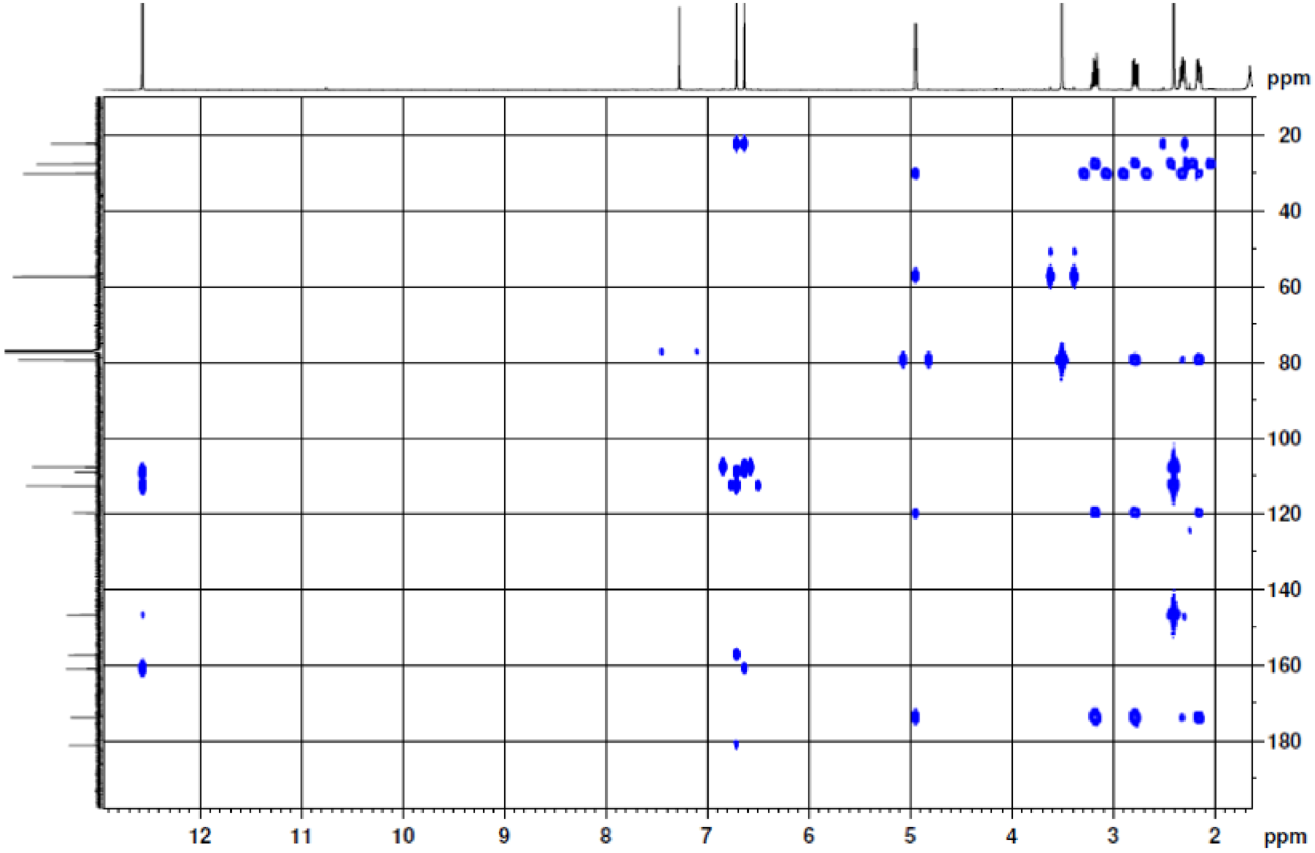

Figure S27. NOESY spectrum (600 MHz, $CDCl_3$) of citrinolactone D.

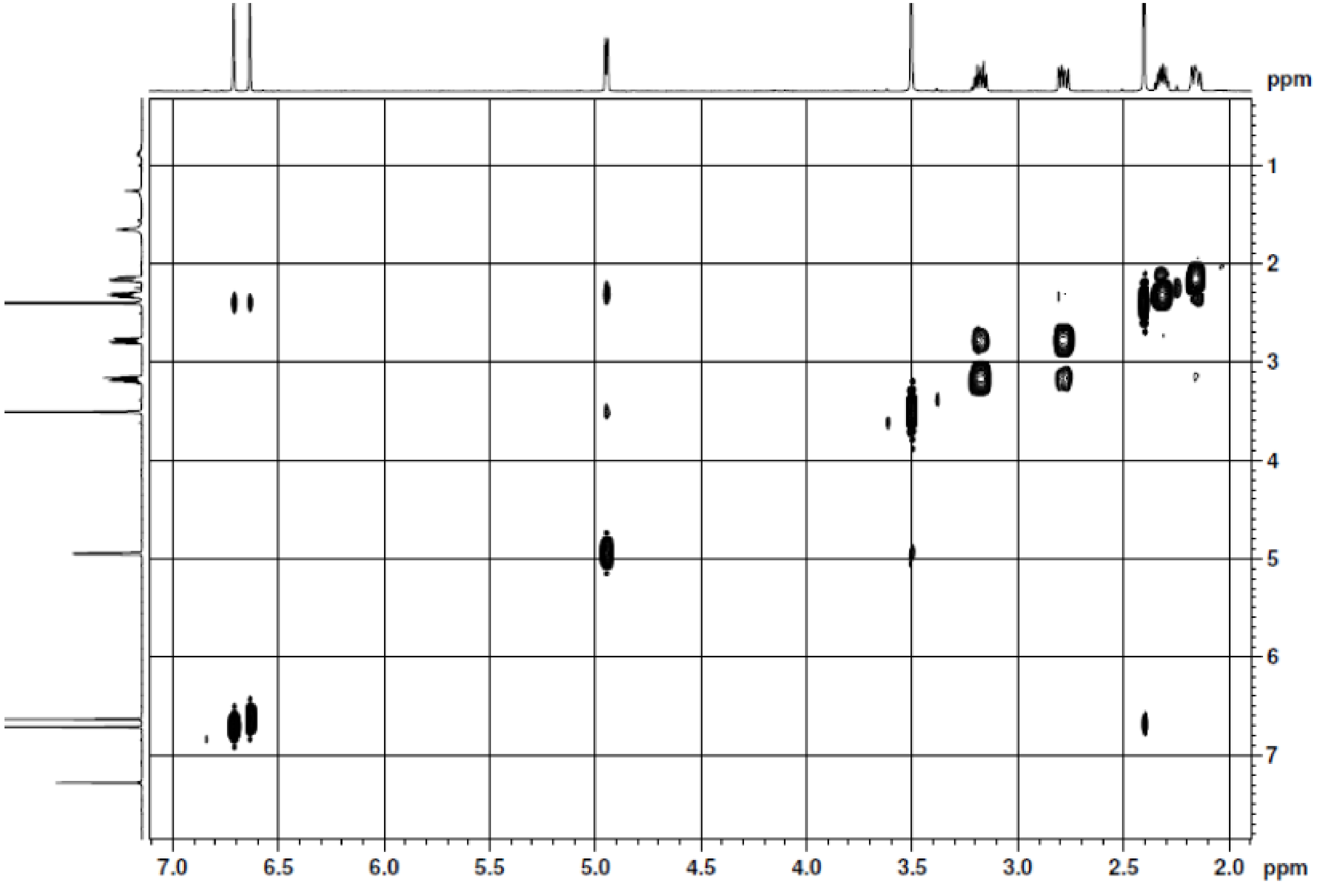

Figure S28. HRESIMS spectrum of citrinolactone D.

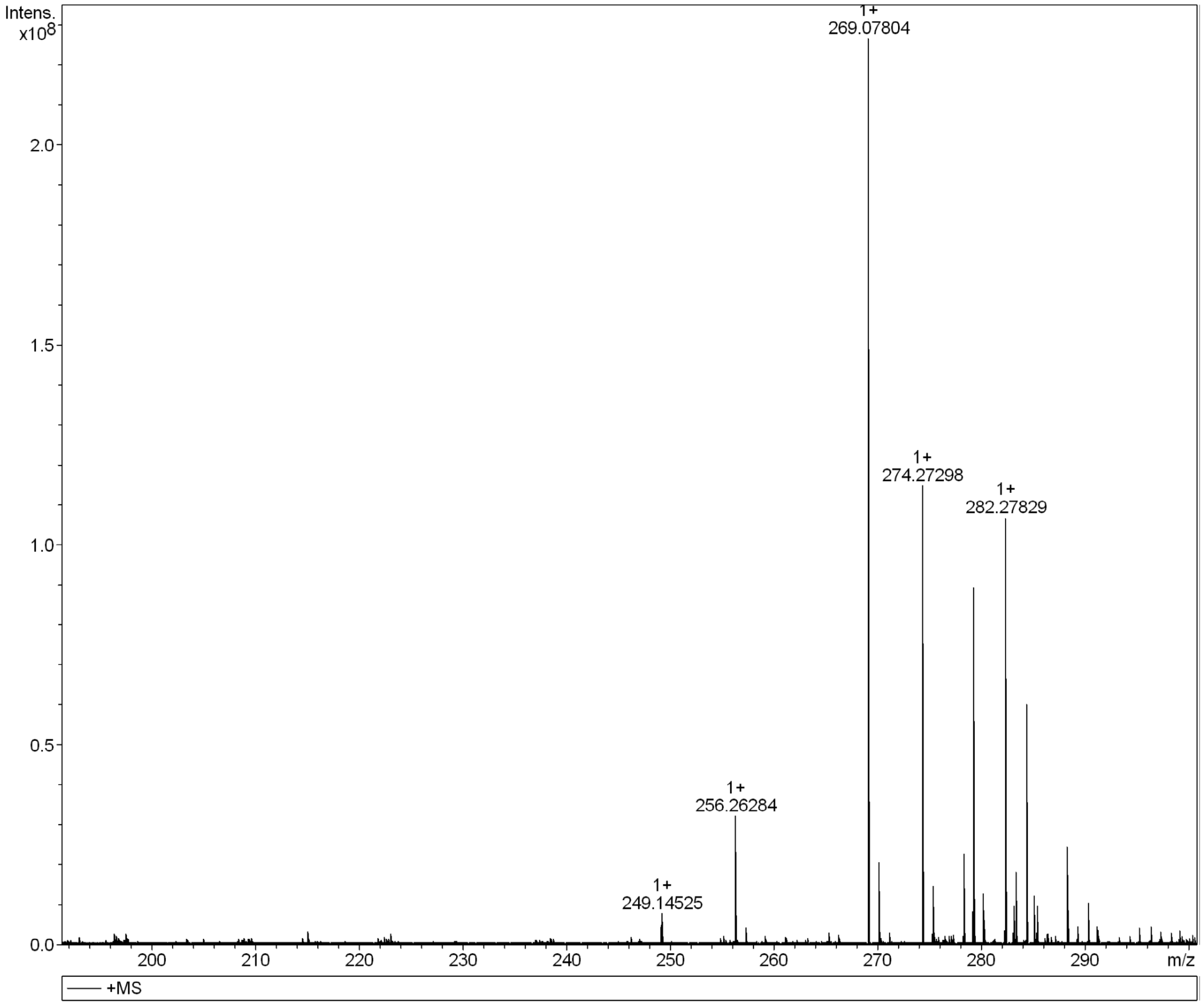

Supplement: Supplementary file 1 [file molecules-18-05723-s001.pdf]
